# Rab37 mediates trafficking and membrane presentation of PD-1 to sustain T cell exhaustion in lung cancer

**DOI:** 10.1186/s12929-024-01009-6

**Published:** 2024-02-07

**Authors:** Wan-Ting Kuo, I-Ying Kuo, Hung-Chia Hsieh, Ssu-Ting Wu, Wu-Chou Su, Yi-Ching Wang

**Affiliations:** 1 Department of Pharmacology, College of Medicine, National Cheng Kung University, No. 1, University Road, Tainan, 701 Taiwan; 2 Department of Biotechnology, College of Biomedical Science, Kaohsiung Medical University, Kaohsiung, Taiwan; 3 Institute of Basic Medicine, College of Medicine, National Cheng Kung University, Tainan, Taiwan; 4 Division of Oncology, Department of Internal Medicine, College of Medicine, National Cheng Kung University, Tainan, Taiwan

**Keywords:** PD-1, Rab37, Membrane trafficking, T cell, Lung cancer

## Abstract

**Background:**

Programmed cell death protein 1 (PD-1) is an immune checkpoint receptor expressed on the surface of T cells. High expression of PD-1 leads to T-cell dysfunction in the tumor microenvironment (TME). However, the mechanism of intracellular trafficking and plasma membrane presentation of PD-1 remains unclear.

**Methods:**

Multiple databases of lung cancer patients were integratively analyzed to screen Rab proteins and potential immune-related signaling pathways. Imaging and various biochemical assays were performed in Jurkat T cells, splenocytes, and human peripheral blood mononuclear cells (PBMCs). *Rab37* knockout mice and specimens of lung cancer patients were used to validate the concept.

**Results:**

Here, we identify novel mechanisms of intracellular trafficking and plasma membrane presentation of PD-1 mediated by Rab37 small GTPase to sustain T cell exhaustion, thereby leading to poor patient outcome. PD-1 colocalized with Rab37-specific vesicles of T cells in a GTP-dependent manner whereby Rab37 mediated dynamic trafficking and membrane presentation of PD-1. However, glycosylation mutant PD-1 delayed cargo recruitment to the Rab37 vesicles, thus stalling membrane presentation. Notably, T cell proliferation and activity were upregulated in tumor-infiltrating T cells from the tumor-bearing *Rab37* knockout mice compared to those from wild type. Clinically, the multiplex immunofluorescence-immunohistochemical assay indicated that patients with high Rab37^+^/PD-1^+^/TIM3^+^/CD8^+^ tumor infiltrating T cell profile correlated with advanced tumor stages and poor overall survival. Moreover, human PBMCs from patients demonstrated high expression of Rab37, which positively correlated with elevated levels of PD-1^+^ and TIM3^+^ in CD8^+^ T cells exhibiting reduced tumoricidal activity.

**Conclusions:**

Our results provide the first evidence that Rab37 small GTPase mediates trafficking and membrane presentation of PD-1 to sustain T cell exhaustion, and the tumor promoting function of Rab37/PD-1 axis in T cells of TME in lung cancer. The expression profile of Rab37^high^/PD-1^high^/TIM3^high^ in tumor-infiltrating CD8^+^ T cells is a biomarker for poor prognosis in lung cancer patients.

**Supplementary Information:**

The online version contains supplementary material available at 10.1186/s12929-024-01009-6.

## Introduction

T cell exhaustion is the most important mechanism of immunosuppression in the tumor microenvironment (TME). The term ‘exhaustion’ is mainly used to refer to effector T cells with reduced capability of cytokine secretion, cytotoxicity, proliferation, and increased expression of inhibitory receptors [1]. These hypofunctional T cells with elevated expression of inhibitory receptors fail to produce IFN-γ, TNF-α, granzyme B, and perforin to eliminate tumor cells. Likewise, intrinsic signaling of T cell expansion is attenuated due to upregulation of inhibitory receptors. Therefore, immune checkpoint blockade (ICB) therapies targeting inhibitory receptors can successfully rejuvenate hypofunctional T cells by blocking inhibitory receptor-mediated dephosphorylation of signaling cascade downstream of T cell activation to suppress tumor development in cancer patients [2, 3]. However, a proportion of patients who initially responded effectively to therapy had relapsed and progressed after a period of ICB therapy. Various factors can confer resistance to ICB such as upregulation of additional coinhibitory receptors. Several studies revealed that increased coexpression and sustainability of multiple immune checkpoints such as programmed cell death 1 (PD-1), TIM3, CTLA-4, and LAG-3 can facilitate severe exhaustion of T cells [4–6]. Therefore, it is worth exploring the mechanism of upregulation of inhibitory receptors in T cells.

PD-1 is a transmembrane inhibitory receptor expressed on the surface of activated T cells. PD-1 ligation to its co-inhibitory ligands, program cell death ligand 1/2 (PD-L1/PD-L2) blunts effector T cell activation, which leads to physiologic limitation of immune responses and thereby averts autoimmunity [7]. Within the TME, tumor-specific T cells expressing high levels of surface PD-1 that could be engaged by its ligand PD-L1 expressed in tumors. PD-1/PD-L1 engagement induces the phosphorylation of the immunoreceptor tyrosine-based inhibitory motif (ITIM) and immunoreceptor tyrosine-based switch motif (ITSM) of PD-1, which in turn recruits and activates Src homology region 2 domain-containing phosphatases (SHPs) to attenuate the phosphorylation-dependent signaling cascades downstream of the T cell receptor (TCR) and the co-stimulatory receptor CD28. PD-1/PD-L1 engagement suppresses cellular functions such as activation, proliferation, metabolic regulation, cytotoxicity, and cytokine production, and thus promotes T cell differentiation into exhausted T cells [8]. It is important to investigate how PD-1 is regulated in T cells during cancer progression.

In general, intracellular level of PD-1 is controlled at several levels including transcriptional and post-translational regulations. For example, nuclear factor of activated T-cell (NFAT), a transcriptional activator, translocates into the nucleus to drive PD-1 coding gene *Pdcd1* expression [9], while B lymphocyte-induced maturation protein 1 (Blimp-1) binding results in the eviction of NFAT from *Pdcd1* locus [10]. Additionally, post-translational modifications of PD-1 such as ubiquitination, glycosylation, acetylation and palmitoylation, also have been extensively studied for protein stability control [11, 12]. For instance, the N-linked glycosylation of PD-1 (gPD-1) proteins at N49, N58, N74 and N116 are critical for PD-1 to maintain its protein stability leading to T cell exhaustion mediated by interaction with PD-L1 [13]. However, the regulation at the trafficking process of intracellular PD-1 after protein synthesis and eventually plasma membrane (PM) presentation has never been identified.

Rab small GTPases play an important role in protein trafficking and some of them regulate cancer progression [14]. Recent study indicated that palmitoylation of PD-1 facilitates interaction with Rab11 for its storage in recycling endosomes to maintain its protein stability [15]. In addition, reduced level of surface PD-1 and increased tumoricidal activity are observed in splenic CD8 T cells derived from Rab37 knockout mice [16]. Rab37 protein mediates vesicle trafficking and protein transport from *trans*-Golgi network to PM or extracellular compartment [17, 18]. These findings suggest that T cell-intrinsic Rab37 may promote T cell dysfunction through sustaining PD-1 membrane presentation.

To determine the regulatory mechanisms of PD-1 intracellular trafficking and PM presentation, we provide first trafficking mode of PD-1 mediated by Rab37 small GTPase. Of note, sustainable PD-1 PM presentation by Rab37 overexpression downregulated T cell proliferation and cytotoxicity in vitro and in vivo. Clinically, patients with Rab37^+^/PD-1^+^/TIM3^+^/CD8^+^ tumor-infiltrating T cells expression profile correlate with poor outcome in lung cancer model. Our findings shed light on Rab37-induced T cell exhaustion through intracellular trafficking and PM presentation of PD-1 with clinical implication for patient evaluation with ICB therapy.

## Materials and methods

### CD8^+^ T cells isolation and activation

Splenocytes derived from wild-type (WT) and *Rab37* knockout (KO) mice were obtained by mashing the spleen and removing erythrocytes using red blood cells lysis buffer. CD8^+^ T cells were isolated using mouse CD8 T lymphocyte enrichment set (BD Bioscience, East Rutherford, NJ, United States). Purified CD8^+^ T cells were activated by either 100 ng/ml of phorbol 12-myristate 13-acetate (PMA, Sigma-Aldrich, Louis City, MO, United States) plus 100 ng/ml of Ionomycin (Io, Sigma-Aldrich) or 2 μg/ml anti-CD3 together with 2 μg/ml anti-CD28 antibodies (BD Bioscience) for 24 or 48 h. T cells with or without treatment with conditioned medium (CM) derived from cancer cells were collected and analyzed by immunofluorescence and flow cytometry.

### Cell lines and culture conditions

Human Jurkat T cells, THP-1 monocyte, and lung cancer cell line H460-Luc stably expressing luciferase gene were maintained in RPMI (Gibco, Los Angeles, CA, United States) with 10% Fetal Bovine Serum (FBS, Gibco), 1% penicillin/streptomycin (Gibco), and 1% sodium pyruvate (Gibco) and cultured at 37 °C with 5% CO_2_ in air. Human lung cancer cell line H1299 was maintained in DMEM (Gibco) supplemented with 10% FBS and 1% penicillin/streptomycin.

### Plasmids, siRNAs and transfection

The siRab37 were purchased from Dharmacon (siGENOME Human RAB37 siRNA, SMARTPool Cat. M-008933-02-0020). A mixture of 4 siRNA (siRAB37-D-008933-01: UCACUGAGAUUCAUGAGUA; siRAB37-D-008933-02: UGGCAUGAAUGUGGAGUUA; siRAB37-D-008933-04: GAUCCGAGACUAUGUAGAG; siRAB37-D-008933-05: GCGGAUAUGAGCAGCGAAA) provided as a single reagent guaranteed to silence target gene expression when used under optimal delivery conditions. A total of 20 nM of siGENOME Human RAB37 siRNA were transfected into Jurkat cells by TransIT®-LT1 (Mirus Part# MIR2304). Human Rab37-WT, GTP-bound active mutant Q89L, and GDP-bound dominant negative mutant T43N pcDNA3.1-His-V5 plasmids were generated as previously described [16]. pCMV3-PDCD-1-GFPspark (human) were purchased from Sino Biological Inc (Beijing, China) and cloned into the pcDNA3.1-His-V5 vector. Plasmid transfection was carried out with IT-J reagent (Mirus Bio) into Jurkat T cells or Turbofect reagent (Thermo Fisher Scientific, Waltham, MA, United States) into 293T cells or COS-1 cells according to manufacturer’s protocol. To generate cells expressing N-linked glycosylation mutants PD-1, pcDNA3.1-GFP-PD-1 was used as a template to generate the PD-1-GFP 1 NQ mutants, that is, N49Q; 2NQ mutants, that is, N49Q, N58Q; 3NQ mutants, that is, N49Q, N58Q, N74Q by performing site-directed mutagenesis. These transfected cells were harvested and applied to in vitro and in vivo studies. A full list of plasmids used are listed in Additional file 1: Table S1.

### Plasma membrane fractionation and Western blot analysis

A total of 8 × 10^7^ Jurkat T cells treated with siRab37 or expressing EV, Rab37-WT, Rab37-Q89L or Rab37-T43N were centrifuged at 100–300×*g* for 5 min at 4 °C and supernatant was discarded. The membrane fractionation was performed according to the manufacturer’s instructions (#ab65400 from Abcam, Cambridge, UK). The pellet is the membrane fraction (plasma membrane proteins), which was dissolved in 0.5% Triton X-100 in PBS and further analyzed by Western blotting. For immunoblotting, the protein was identified by incubating the membrane with primary antibodies, followed by horse radish peroxidase-conjugated secondary antibodies. Immunoreactive proteins were visualized using Western blot chemiluminescence reagent (Millipore, Burlington, Massachusetts, United States) and the signals were detected by Luminescence Readers (FUJI LAS-1000, Fujioka, Japan).

### Vesicle isolation and immunoprecipitation

Vesicle isolation protocol was modified from Kuo’s report [16]. Jurkat T cells (2 × 10^7^) expressing His-V5-tagged Rab37 were resolved in 300 μl of 200 mM sucrose and gently sonicated. The supernatants were obtained by centrifugation (3000×*g* for 10 min at 4 °C). Vesicles were enriched from supernatants by high-speed centrifugation (30,000×*g* for 60 min at 4 °C) using a 40-Ti rotor (Beckman, Coulter, CA, United States). The vesicles-containing solution was incubated with anti-V5 antibody to isolate Rab37-specific vesicles and the cargos in vesicles were analyzed by Western blot using the indicated antibodies.

### Total internal reflection fluorescence (TIRF) microscopy

The protocol was modified from Kuo’s report [16]. In brief, 293T (5 × 10^5^) cells were seeded in 3.5 cm glass bottom dish. After 48 h, cells were transfected with RFP-Rab37 or GFP-PD-1 for 18 h. TIRF microscopy system was built on an inverted microscopy Olympus IX81 (Olympus, Tokyo, Japan) equipped with a high sensitivity EMCCD Camera (iXOn3897, Andor technology, New York, United States) and a UPON 100X oil objective lens (NA = 1.49, Olympus) to capture 100–200 nm images below the plasma membrane interface. We defined each green fluorescence spot as a cargo-containing vesicle and trafficking by Rab37 in cells, and then tracked each vesicle trafficking distance with trackIT software (Olympus). The cutoff length of moving vesicle was 3 μm. The vesicle trafficking event was measured as the ratio of moving vesicles to the total vesicles in cells. A total of at least 20 moving vesicles per cell were tracked and 6 cells were scored.

### Real-time live confocal fluorescence microscopy

The time-lapse experiments were captured by Olympus FV3000 confocal microscope and used FV31S-SW software (Olympus) to analyze the co-localization of Rab37 and PD-1 in Rab37-WT 293T cells. Rab37-RFP WT and PD-1-GFP were transfected and expressed for 16–18 h in 293T cells. The Rab37-RFP and PD-1-GFP signals in 293 T cells were recorded through real-time live image and video through the 100× lens of the microscope. Quantification was done using Image J software.

### Immunogold electron microscopy

For immunogold electron microscopy analysis, Jurkat T cells were fixed, sectioned, and immersed in H_2_O_2_ for 10 min, blocked with 1% BSA for 1 h and then incubated with indicated antibodies and protein A coupled to 10 nm or 20 nm gold particles. Sections were post-stained with uranyl acetate and lead citrate, and then examined with a transmission electron microscope JEM-1400 (JEOL, Tokyo, Japan).

### Carboxyfluorescein succinimidyl ester (CFSE) cell proliferation assay

To detect proliferation of splenocytes co-cultured with cancer cells, T cells (1 × 10^6^) were labeled with 5 µM CFSE dye (Thermo Fisher Scientific) in PBS and incubated at 37 °C for 20 min. After 20 min, cells were diluted with 5 volume of complete RPMI medium and incubated for 5 min. Cells were centrifuged for 5 min at 300×*g* and the cell pellet was resuspended and cultured with cancer cells in 12-well plate for 48 h. The ratio of splenocytes to cancer cells in co-culture was 10:1.

### Flow cytometry

Splenocytes or Lewis lung carcinoma (LLC) tumors from *Rab37* KO or *Rab37* WT mice were stained with anti-mouse CD8, CD4, CD107a, Ki67, TIM3, TNF-α, CD25, and PD-1. Human peripheral blood mononuclear cells (PBMCs) from lung cancer patients or healthy donors were stained with anti-human CD8, Granzyme B (GzmB), IFN-γ, TIM3, and PD-1 antibodies. All antibodies were diluted 1:200 with staining buffer. Cells were incubated on ice for 30 min, washed and fixed with 2% paraformaldehyde then kept in staining buffer. After cell surface staining (CD8, CD4, TIM3, CD25, and PD-1), intracellular Ki67, GzmB, IFN-γ, TNF-α, and CD107a were stained with antibodies, which was diluted 1:200 with permeabilized buffer. A full list of antibodies used are listed in Additional file 1: Table S2.

### Immunofluorescence (IF) and immunofluorescence-immunohistochemistry (IF-IHC) assay and confocal microscopy analysis

The process was performed according to the manufacturer’s instructions (Opal stain kit #NEL810001KT, Akoya Biosciences, Marlborough, MA, United States) and modified from Kuo’s report [16]. In brief, a total of 7 × 10^3^ splenic T cells or Jurkat T cells were prepared in 100 μl complete RPMI and concentrated on the slide using cytospin at 600 rpm for 5 min and fixed with 1% formaldehyde for 20 min. After antigen retrieval, samples were covered with primary antibody and incubated at 4 °C overnight or 1 h, depending on antibody. The next day, samples were incubated with polymer HRP of mouse or rabbit species for 10 min and Opal fluorophore for 10 min at room temperature. For staining of another primary antibody, the microwave steps were repeated. The images were captured by Olympus FV3000 confocal microscope and FV31S-SW software (Olympus) was used to analyze the co-localization of Rab37 with PD-1 in WT or *Rab37* KO splenic T cells or EV, Rab37-WT, Rab37-Q89L or Rab37-T43N expressing Jurkat T cells. For Multiplex IF-IHC staining in samples from animal or cancer patients, Opal stain kit (Akoya Biosciences) was used and staining was performed according to the manufacturer’s instruction. The slides were stained with Rab37, CD8, TIM3, PD-1, or LAG3 primary antibody at 4 °C overnight. After Opal staining process, DAPI was applied for nuclei staining. Multiplex IF-IHC were conducted to examine the localization of Rab37, CD8^+^ T cells, CD4^+^ T cells, PD-1, TIM3, or LAG3 in allografts from mice or tumor specimens from cancer patients. Whole slides were scanned at 10× magnification (OLYMPUS cellSens) for visualization of the tumor, three non-overlapping regions of interest (ROIs) were selected and scanned at 20× for quantification. The size of the ROI was 200 × 200 µm (0.04 mm^2^) in allograft from mice, and 200 × 200 µm (0.04 mm^2^) in clinical patients’ tissue slides. The detailed antibodies conditions are listed in Additional file 1: Table S2.

### Allograft tumor growth in vivo

Whole body systemic *Rab37* KO in the C57BL/6 background mice were established in previous study [16]. A total of 5 × 10^5^ LLC cells were subcutaneously inoculated into *Rab37* KO mice and WT mice. All mice were sacrificed at the end of endpoint of experiment. Mice genetically deficient in the Rag1 gene (*Rag1* KO) on the C57BL/6 background were kindly provided by Dr. Ping-Ning Hsu (National Taiwan University, Taipei, Taiwan). A total of 5 × 10^5^ LLC cells were mixed with splenic CD8 T cells derived from WT or *Rab37* KO mice and then subcutaneously injected into flank of WT or *Rag1* KO C57BL/6 mice. For subcutaneous model, tumor volume was calculated at day 8 and once every 3 days using the equation V = (a^2^ × b)/2 during observation. All animal experiments were performed in compliance with National Cheng Kung University institutional guidelines for use and care of animals (Permit Numbers: #112004).

### Tumor specimens and human peripheral blood mononuclear cells of lung cancer patients

A total of 60 surgically resected lung cancer patients were recruited from National Cheng Kung University Hospital after obtaining appropriate institutional review board permission (#B-ER-110-104) and informed consent from the patients. These patients were analyzed for IF-IHC of tumor-infiltrating T cells in their surgical specimens. Overall survival was calculated from the day of surgery to the date of death or the last follow-up. Tumor typing and disease staging were performed according to the World Health Organization classification and the TNM classification system, respectively. Information on the age, sex, and smoking history of the patients were obtained from hospital records. Additionally, a total of 12 PBMCs were collected from three healthy donors and nine lung cancer patients without ICB therapy recruited from National Cheng Kung University Hospital after obtaining appropriate institutional review board permission (#B-ER-110-104) and informed consent from the patients (Additional file 1: Table S3). All PBMCs were cultured in RPMI supplemented with 10% FBS and 1% penicillin/streptomycin (Gibco) at 37 °C with 5% CO_2_ in air. Additionally, PBMCs were treated with 1 μg/ml anti-CD3/CD28 antibody plus 10 ng/ml IL-2 for 7 days. After 7 days, all stimulated PBMCs were analyzed for ex vivo T cell functionality. PBMCs were co-cultured with H460-Luc lung cancer cells in 96-well plate for 48 h. The ratio of PBMCs to cancer cells in co-culture was 3:1.

### Statistical analysis

Cell studies were conducted in three independent experiments unless indicated otherwise. Data represented mean ± S.D. using two-tailed Student’s t test. The statistical analyses of intratumoral Rab37, PD-1, TIM3 or CD8 expression level with patients’ survival time were performed using Statistical Package for the Social Sciences version 26.0 (SPSS Inc., Headquarters Chicago, IL, United States). Overall survival and progression-free survival curves were calculated according to the Kaplan–Meier method by the log-rank test. Correlations were examined using Pearson’s correlation test. Receiver operating characteristic (ROC) curve analysis was used to determine the value of PD-1^+^/TIM3^+^/CD8^+^ T cells and PD-1^+^/TIM3^+^/CD8^+^/Rab37^+^ T cells in lung cancer patients. The parameters including sensitivity, specificity, and area under the ROC curve (AUC) with 95% confidence interval (CI) were also evaluated. The level of statistical significance was taken as *P* value, *P < 0.05; **P < 0.01; ***P < 0.001.

## Results

### PD-1 expression level positively correlates with Rab37 in T cells

**Fig. 1:**
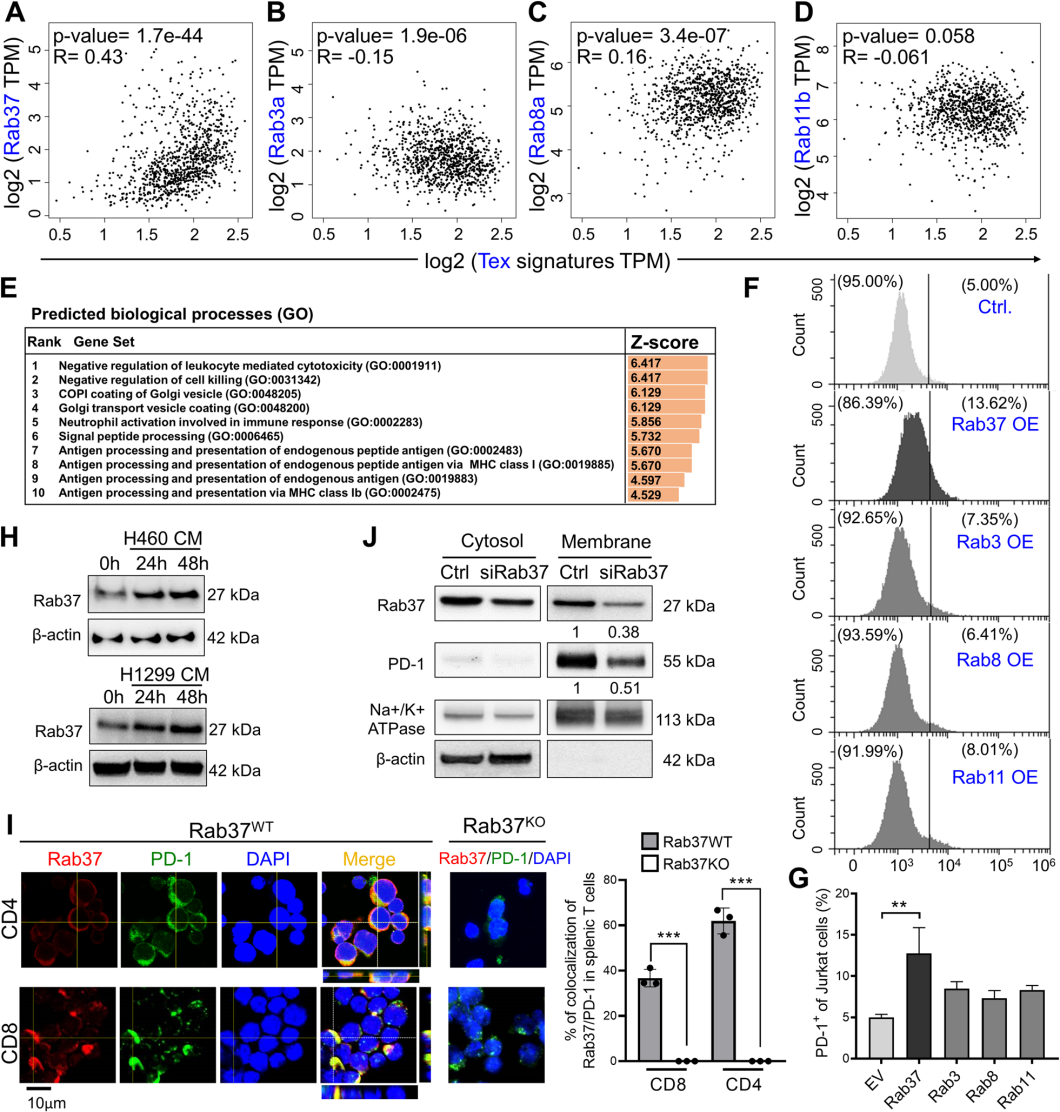
PD-1 expression level positively correlates with Rab37 in T cells. **A**–**D** The association of exhaustion T cell (Tex) gene signatures with Rab37, Rab3a, Rab8a, and Rab11b in lung cancer patients using GEPIA database. Pearson correlation coefficient, R square and P value are shown. **E** The enrichment of Rab37 in predicted biological processes using ARCHS4 website. **F**, **G** Flow cytometry (**F**) and quantification (**G**) was performed to determine PD-1^+^ in Jurkat T cells overexpressing Rab37, Rab3a, Rab8a, or Rab11b. **H** Jurkat T cells were incubated with CM from H460 or H1299 cancer cells for the indicated time. The expression of Rab37 and β-actin protein was detected by Western blot. **I** Confocal images (left) and quantitative results (right) of Rab37 and PD-1 in WT and *Rab37* KO splenic CD4^+^ and CD8^+^ T cells treated with PMA/Io for 24 h. Z-score images of lines indicated in the merge panel are shown. Rab37 (red); PD-1 (green). Blue signals represented DAPI (nucleus staining). Scale bar: 10 μm. The colocalization of PD-1 and Rab37 in splenic T cells was determined. **J** Jurkat T cells were transfected with siRab37-oligos for 24 h with PMA/Io treatment. Cells were then subjected to cytosolic and plasma membrane fractionation. The band intensities were quantified, and the normalized fold changes are indicated below the blots. The data are shown as the mean ± S.D. P values were determined by two-tailed Student’s t test. *P < 0.05, **P < 0.01, ***P < 0.001

To understand the mechanisms of intracellular trafficking and PM presentation of PD-1 by Rab GTPases, we first identified the relevance of PD-1 expression with Rab3a, Rab8a, Rab11b and Rab37 proteins which regulate protein transport from the Golgi network to the PM. We found that exhaustion T cell (Tex) gene signatures showed a stronger positive correlation with *Rab37* compared to *Rab3a*, *Rab8a* or *Rab11b* in patients with lung adenocarcinoma (n = 483) and lung squamous cell carcinoma (n = 486) datasets (Fig. 1A–D) from the Gene Expression Profiling Interactive Analysis (GEPIA) website [19]. In addition, *Pdcd1* expression showed strong positive correlation with Rab37 expression in lung cancer patients (Additional file 1: Fig. S1A–D), while it was found to be weakly associated with *Rab37* in normal tissues (n = 109) (Additional file 1: Fig. S1E–H). Next, we input Rab37 gene into ARCHS4 website (https://maayanlab.cloud/archs4/download.html) to obtain *Rab37* gene landing page containing predicted biological processes (GO) based on correlations with *Rab37* gene assigned to GO categories. The GO Term showed that Rab37 expression was enriched in negative regulation of leukocyte-mediated cytotoxicity and cell killing pathways (Fig. 1E). To determine whether the PM presentation of PD-1 was mainly mediated by Rab37, we ectopically expressed Rab37, Rab3a, Rab8a or Rab11b in Jurkat T cells for flow cytometry analyses of PD-1^+^ membrane presentation (Additional file 1: Fig. S1I). The data showed that only Rab37 expressing Jurkat T cells presented substantial level of cell surface PD-1 (Fig. 1F, G). In addition, in response to lung cancer cells (H460 and H1299 cells) conditioned medium (CM) treatment, Rab37 was upregulated in Jurkat T cells at 24 h and 48 h (Fig. 1H). These database analyses and experimental results suggested that upregulation of Rab37 is involved in PD-1 expression and immune suppression in the TME of lung cancer.

Next, we performed confocal immunofluorescence (IF) with z score presentation to observe the localization of Rab37 and PD-1 in ex vivo stimulated splenocytes. The merge panel of confocal IF images and their quantification results demonstrated the intracellular colocalization of Rab37 and PD-1 in CD4^+^ and CD8^+^ splenic T cells from WT mice, but not in those from *Rab37* KO mice (Fig. 1I). Accordingly, Rab37-mediated PD-1 membrane presentation was validated by the immunoblotting assay of extracted membrane proteins from Jurkat T cells treated with or without siRab37 oligo. Our results showed that silencing Rab37 reduced substantial level of surface PD-1 on the plasma membrane compared with control (Fig. 1J). Together, these results suggest that Rab37 mediates PD-1 membrane presentation in splenic T cells and Jurkat T cells.

### Rab37 mediates PD-1 intracellular trafficking and PM presentation through a GTP-dependent manner in T cells

**Fig. 2:**
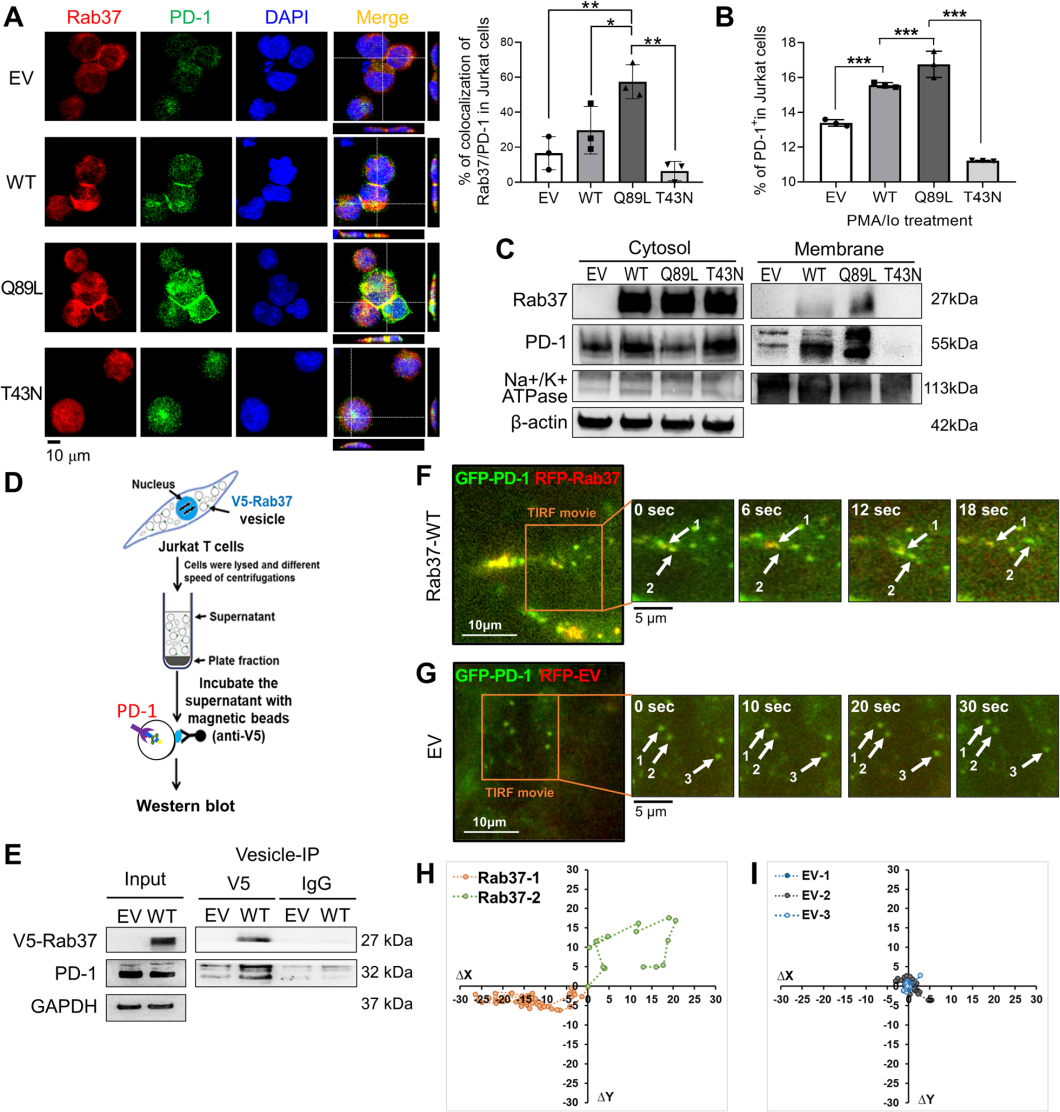
Rab37 mediates PD-1 PM presentation in a GTP-dependent manner. **A** Confocal images (left) and quantitative results (upper right) of Rab37 and PD-1 in EV, Rab37-WT, -Q89L and -T43N Jurkat T cells after transfection for 24 h with PMA/Io treatment. Rab37 (red); PD-1 (green). Blue signals represented DAPI (nucleus staining). Z-score images of lines indicated in the merge panel are shown. Scale bar: 10 μm. The colocalization of PD-1 and Rab37 in Jurkat T cells was determined (upper right). **B** PD-1 PM presentation level in EV, Rab37-WT, Q89L and -T43N Jurkat T cells with PMA/Io treated for 24 h measured by flow cytometry. **C** Jurkat T cells were transfected with EV, Rab37-WT, -Q89L or -T43N plasmid for 24 h with PMA/Io treatment. Cells were then subjected to cytosolic and membrane fractionation and immunoblotting. **D** Vesicles of EV and V5-tagged Rab37-WT (V5-Rab37) Jurkat T cells were treated with PMA/Io for 24 h and then enriched by serial centrifugations. **E** The vesicle-containing supernatants were immunoprecipitated with anti-V5 beads and lysate were blotted for V5-Rab37 and PD-1. Immunoblots confirmed that PD-1 proteins were enriched in Rab37-specific vesicles. **F**, **G** The dynamics of Rab37-regulated PD-1 trafficking by TIRF assay in live cells. Selected frames from time-lapse images of RFP-tagged Rab37 (RFP-Rab37) 293T cells expressing GFP-tagged PD-1 (GFP-PD-1) (**F**) or cells expressing RFP-EV with GFP-PD-1 (**G**). Enlarged images of the boxed areas from Additional file 2: Movie S1, Additional file 3: Movie S2 with time intervals in seconds are shown. Arrow indicated horizontal movement of colocalized RFP-Rab37/GFP-PD-1 puncta. Scale bar: 10 μm. **H**, **I** Migration tracks of vesicle trafficking events and distance in cells expressing Rab37 (**H**) or EV (**I**) were demonstrated by trackIT software. Each colored symbol represents the indicated vesicles in **F** and **G**. The data are shown as the mean ± S.D (n = 3 per group). P values were determined by two-tailed Student’s t test. **P < 0.01, ***P < 0.001

Since GTP bound Rab proteins facilitate vesicle transport, protein trafficking, membrane targeting and fusion of cargo proteins, we investigated whether Rab37 mediated PD-1 membrane presentation through a GTP-dependent manner in T cells. Protein expression and intracellular localization of PD-1 were examined in empty vector (EV), Rab37-WT, GTP-bound active Rab37-Q89L and GDP-bound inactive Rab37-T43N overexpressing Jurkat T cells. The confocal IF staining illustrated that PD-1 protein was upregulated and colocalized with Rab37 in Rab37-WT and Rab37-Q89L Jurkat T cells compared to EV and Rab37-T43N groups after stimulated with PMA/Io (Fig. 2A).

To further investigate the Rab37-mediated cell surface presentation of PD-1 in T cells, we measured the surface PD-1 expression on the PM of EV, Rab37-WT, Rab37-Q89L and Rab37-T43N overexpressing Jurkat T cells by flow cytometry. Consistently, the percentages of cell surface PD-1^+^ in Jurkat T cells were higher in Rab37-WT group and Rab37-Q89L group but lower in Rab37-T43N group, compared to EV group (Fig. 2B). Indeed, results of membrane fractionation assay showed that PD-1 protein in the membrane compartment was upregulated in Rab37-WT and Rab37-Q89L Jurkat T cells, while the membrane signals were almost abolished in Rab37-T43N inactive group (Fig. 2C). Notably, we hypothesized that GTP-bound Rab37 can mediate PD-1 through vesicle trafficking to sustain PM presentation. Thus, we ectopically expressed EV, Rab37-WT, GTP-bound active Rab37-Q89L and GDP-bound inactive Rab37-T43N in Jurkat T cells containing NFAT luciferase reporter and then monitored T cell activation. The results indicated that constitutively active Rab37QL OE Jurkat T cells exerted low NFAT activity (Additional file 1: Fig. S2A), suggesting that GTP-bound Rab37 mediates PM presentation of PD-1 to suppress T cell activation. These results suggest that Rab37 colocalizes with PD-1 and regulates its PM presentation in T cells through a GTP-dependent manner.

To further confirm that PD-1 was a cargo of Rab37-mediated vesicle trafficking, we used V5-tagged antibody to immunoprecipitate Rab37-specific vesicles in EV and Rab37-WT overexpressing Jurkat T cells, and performed immunoblotting to detect PD-1 protein level (Fig. 2D). The vesicle-IP data showed that PD-1 was enriched in Rab37-specific vesicles derived from WT group compared to that from EV group (Fig. 2E). Moreover, immunogold electron microscopy demonstrated the ultrastructural co-localization of Rab37 and PD-1 and showed Rab37-mediated vesicle recruitment for cell surface presentation of PD-1 in the PMA/Io treated Jurkat T cells (enlarged image of the two indicated areas) (Additional file 1: Fig. S2B). These results together suggest that Rab37 colocalizes with PD-1 in the same vesicle compartment to mediate the vesicle trafficking of PD-1.

To visualize the real time effect of Rab37 on PD-1 transportation, total internal reflection fluorescence (TIRF) assay was performed to observe fluorescent-labelled signals in the proximity 200 nm below of the PM. Jurkat T cells are suspension cells and obtaining live imaging of suspension cells is still a challenge, therefore, we used the 293T cells for TIRF live imaging assays. We co-expressed the GFP-tagged PD-1 (GFP-PD-1) and RFP-tagged Rab37 (RFP-Rab37) or RFP-EV in 293T cell and the TIRF images showed that GFP-PD-1 signals appeared and moved rapidly in RFP-Rab37 overexpression cells (Fig. 2F and Additional file 2: Movie S1). However, fluorescent puncta barely moved in 293T cells transfected with GFP-PD-1 and RFP-EV group (Fig. 2G and Additional file 3: Movie S2). The pseudo-colored migration tracks of individual cells were shown in Fig. 2H, I. Moreover, the real-time confocal images of 293T cells showed that yellow colocalization signals rapidly appeared and moved in Rab37-WT cells (Additional file 1: Fig. S2C and Additional file 4: Movie S3), while the fluorescence signals hardly moved in 293T cells transfected with GFP-PD-1 and RFP-EV (Additional file 1: Fig. S2D and Additional file 5: Movie S4). The quantitative results were shown in Additional file 1: Fig. S2E. All together, these imaging and biochemical analyses demonstrate that GTP bound Rab37 mediates the dynamic trafficking movement and PM presentation of PD-1 in T cells.

### Rab37 regulates PD-1 intracellular trafficking and PM presentation depending on PD-1 glycosylation

Glycan structures on newly synthesized proteins are crucial for protein folding, function, and secretion [20, 21]. Previous studies have shown that gPD-1 is critical for PD-1 to maintain its protein stability, membrane presentation on T cells and mediate interaction with its co-inhibitory ligand, PD-L1 [13]. The cargo recruitment of Rab37 occurs on the *trans*-Golgi side, which is also the main organelle of glycosylation. Therefore, we hypothesized that only the gPD-1 with correct glycosylation could be transported by Rab37-specific vesicles.

**Fig. 3:**
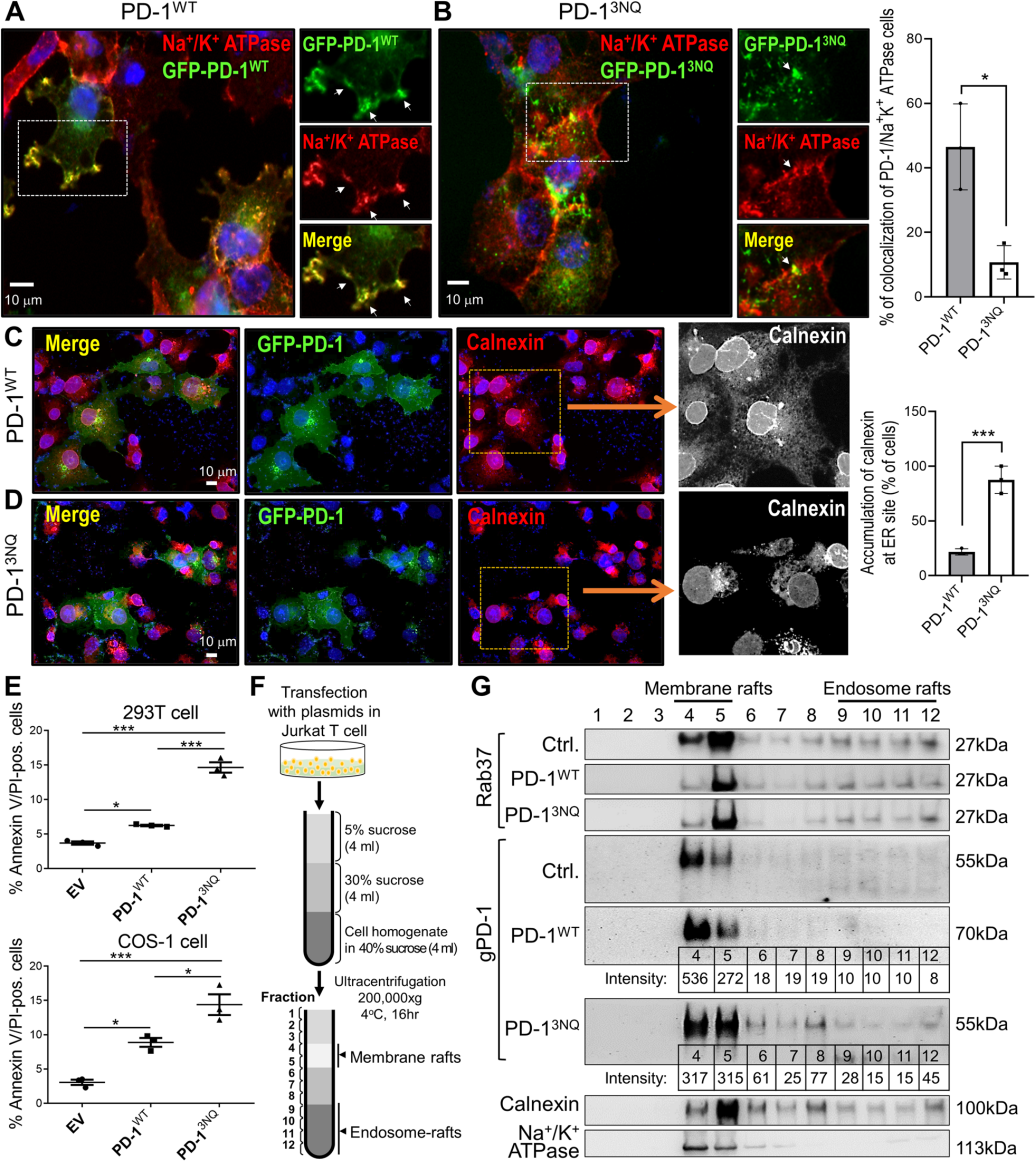
Rab37 regulates glycosylated PD-1 membrane presentation. **A**, **B** Confocal images of PD-1 (green) and Na^+^/K^+^ ATPase (red, PM marker) in COS-1 cells expressing GFP-WT-PD-1 or GFP-3NQ-PD-1. Scale bar: 10 μm. Colocalization of confocal images of PD-1 and Na^+^/K^+^ ATPase was determined (right). **C**, **D** COS-1 cells were transfected with either GFP-WT-PD-1 or GFP-3NQ-PD-1 plasmid followed by immunostaining of Calnexin (red, ER marker). Scale bar: 10 μm. Accumulation of calnexin at ER compartment was determined (right). **E** Annexin V/PI staining was performed at 48 h by flow cytometry in 293T cells and COS-1 cells. **F**, **G** Immunoblot analyses of sucrose gradient fractionations in Jurkat T cells after transfection with either GFP-WT-PD-1 or GFP-3NQ-PD-1 for 18 h. Calnexin and Na^+^/K^+^ ATPase proteins were blotted in addition to Rab37 and gPD-1. Protein intensity of PD-1^WT^ and PD-1^3NQ^ at fractions 4–12 was determined by image J and are indicated below the blots. The data are shown as the mean ± S.D. P values were determined by one-way ANOVA. *P < 0.05, **P < 0.01, ***P < 0.001

To this end, we used site-directed mutagenesis to generate 3NQ-mutant PD-1 expression vector with mutations at N49, N58, and N74. The molecular weight of gPD-1 was decreased when mutation occurred at multiple glycosylation sites (Additional file 1: Fig. S3A). The localization of gPD-1 was investigated by transient transfection of 293T and COS-1 cells with GFP-gPD-1. These cells were chosen for their relatively large cytosolic compartment that enabled the capture of higher-quality images. We first used organelle markers of calnexin, TGN46, EEA1, Rab11b, and Na^+^/K^+^ ATPase to label the ER, Golgi apparatus, early endosome, recycling endosome, and PM respectively, and to examine the cellular distribution of gPD-1 proteins (Additional file 1: Fig. S3B–F). IF images depicted the colocalization patterns of GFP-gPD-1^WT^ with PM marker Na^+^/K^+^ ATPase, while GFP-gPD-1^3NQ^ mutant was mostly localized with ER marker calnexin following 18 h post-transfection (Fig. 3A, B and Additional file 1: Fig. S3B, E). Notably, the staining of calnexin showed a predominantly aggregated phenotype in cytoplasm in GFP-gPD-1^3NQ^ mutant compared with GFP-gPD-1^WT^ group (Fig. 3C, D), suggesting that cell death may be triggered in response to ER stress in GFP-gPD-1^3NQ^ group. Thus, we examined the expression of three major ER stress-related pathways (PERK, ATF6, and IRE1α) by Western blot and cell death by Annexin V-PI staining. As the results shown in Additional file 1: Fig. S3G and Fig. 3E, overexpression of GFP-gPD-1^3NQ^ facilitated activation of PERK signal to promote cell death at 48 h compared with GFP-gPD-1^WT^ group, indicating that GFP-gPD-1^3NQ^ glycosylated mutant accumulated at the ER site leading to ER stress and cell death.

The observation of gPD-1 mutant exhibiting ER stress prompted us to hypothesize that deficiency of N-glycosylation of PD-1 may lead to inhibition of Rab37-mediated PD-1 intracellular trafficking and PM presentation. Therefore, we performed sucrose gradient ultracentrifugation to reveal the distribution of GFP-gPD-1^WT^ and GFP-gPD-1^3NQ^ in the intracellular compartments (Fig. 3F). The quantitative results of immunoblots showed that gPD-1 proteins in the GFP-gPD-1^WT^ group were expressed more in the large membrane compartment (fraction 4) than did the GFP-gPD-1^3NQ^ glycosylated mutant group which was more in the smaller membrane rafts (fraction 5) (Fig. 3G). In addition, the expression pattern of PD-1-3NQ was similar to that of the ER marker calnexin (fractions 6–12), indicating that PD-1-3NQ accumulated at ER compartment. Quantitively, some GFP-gPD-1-3NQ glycosylated mutant proteins still remained in the endosome compartment (fractions 9–12) compared to PD-1-WT, suggesting that the transportation of PD-1-3NQ to the plasma membrane is partially impaired (Fig. 3G). These results suggest that GFP-gPD-1^WT^ proteins exerted fast trafficking and membrane targeting abilities. However, GFP-gPD-1^3NQ^ glycosylated mutant proteins remained in the ER-to-Golgi endosomes and were delayed in trafficking and fusion with the PM compartment. Taken together, these pieces of evidence suggests that glycosylation of PD-1 is important for Rab37-mediated PD-1 intracellular trafficking and membrane presentation in T cells.

### PD-1 sustainable PM presentation mediated by overexpression of Rab37 inhibits T cell activation in tumor-infiltrating cells in vivo and human peripheral blood mononuclear cells

**Fig. 4:**
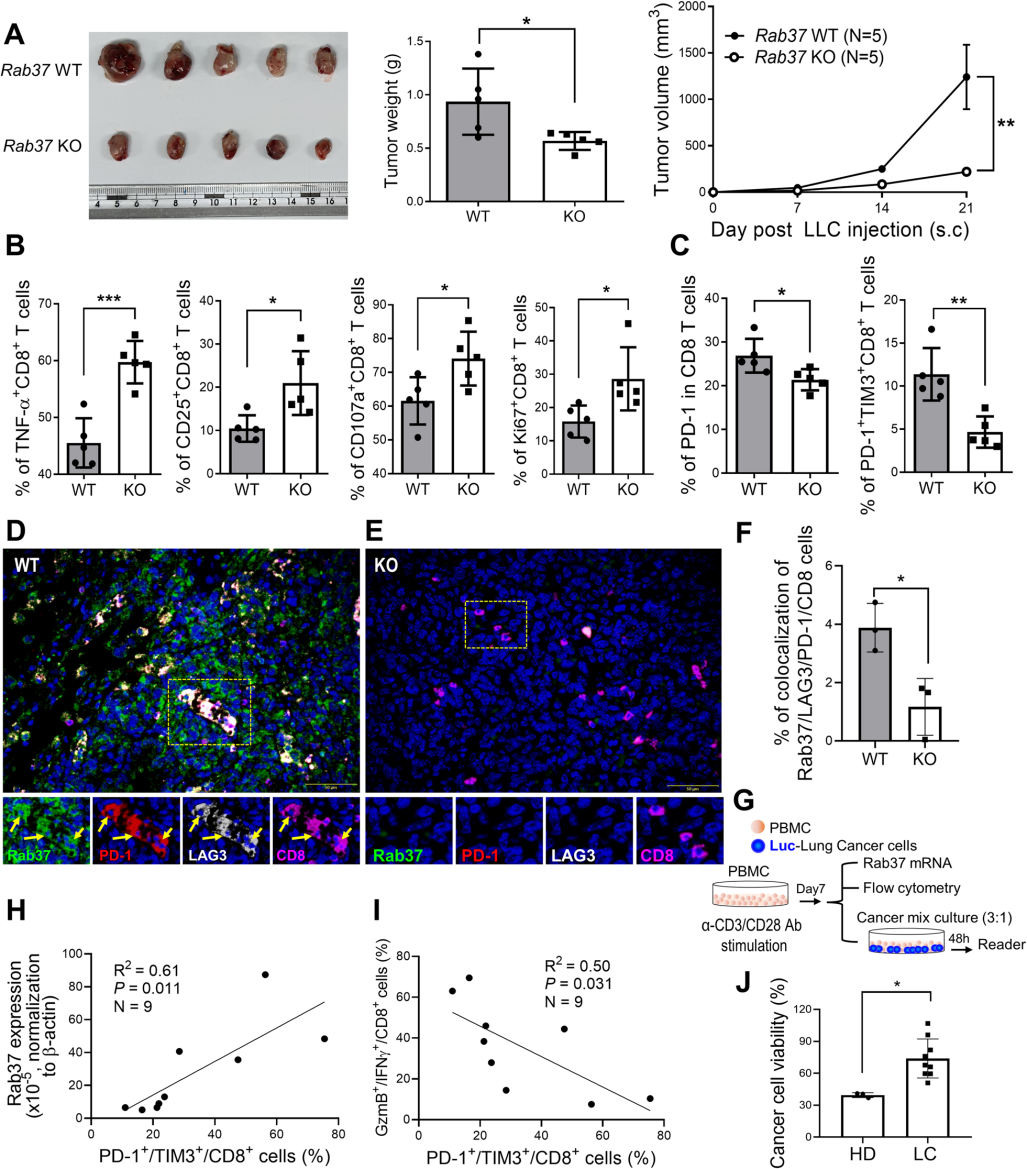
Rab37 facilitates PD-1 PM presentation to suppress activation of T cells. **A** Tumor growth of the subcutaneous syngenetic mouse LLC allograft (5 × 10^5^ cells inoculation) in *Rab37* WT and KO C57BL/6 mice. Tumor size, weight, and volume are shown as indicated. **B**, **C** Tumor-infiltrating CD8^+^ T cells of endpoint tumor derived from *Rab37* WT and KO mice on day 21 were analyzed by flow cytometry for various T cell functional markers as indicated. **D**, **E** Paraffin wax-embedded LLC tumor sections from *Rab37* WT (**D**) and KO mice (**E**) were stained with Rab37 (green), PD-1 (red), LAG3 (white) and CD8 (purple). Blue signals represented DAPI (nucleus staining). Enlarged panels of the inset in the merged image are shown below. Colocalization of Rab37, PD-1 and LAG3 in CD8^+^ T cells were detected by merging the mono-staining pictures. **F** The quantification of colocalization of Rab37, PD-1 and LAG3 in tumor-infiltrating CD8^+^ T cells was determined. **G**–**J** Human PBMCs collected from healthy donors and lung cancer patients were cultured for 7 days followed by anti-CD3/28 antibodies and IL-2 treatment for analyzing *Rab37* mRNA expression, GzmB/IFN-γ expression and PD-1/TIM3 expression then were co-cultured with H460-Luc cells for cancer cell viability (**G**). A total of 9 lung cancer patient samples were analyzed for *Rab37* mRNA expression, GzmB/IFN-γ expression and PD-1/TIM3 expression (**H**, **I**). The cancer cell viability was demonstrated for PBMCs derived from healthy donors (HD) and lung cancer patients (LC) (**J**). The data are shown as the mean ± S.D. *P* values were determined by one-way ANOVA or two-tailed Student’s t test. *P < 0.05, **P < 0.01, ***P < 0.001

To validate the impact of Rab37-mediated PD-1 PM presentation on the functions of T cells in the TME, we performed subcutaneous tumor model in whole-body *Rab37* KO and WT mice and then examined the functional signature of tumor-infiltrating T cells. To exclude the physiological concern of Rab37 deficiency in mice, we firstly examined the serum biochemical parameters and histology of major organs from *Rab37* KO and WT subcutaneous tumor-bearing mice. The hallmark of liver function (GOT/AST and GPT/ALT), kidney function (BUN, creatinine), and normal physiology (albumin) showed no significant difference between *Rab37* KO and WT mice (Additional file 1: Fig. S5A). In addition, histology of kidney, liver, lung, and spleen showed no significant difference between *Rab37* KO and WT mice (Additional file 1: Fig. S5B), suggesting that depletion of Rab37 had no significant side effects compared to WT mice. The results demonstrated that LLC tumor growth in *Rab37* KO mice was inhibited compared to that in WT mice (Fig. 4A). In addition, the results of functional assay indicated an increase in cytokine production (TNF-α), proliferation (Ki67), cytotoxicity (CD107a), and activation marker (CD25) in tumor-infiltrating CD8^+^ T cells of *Rab37* KO group compared to those in tumors of WT mice (Fig. 4B). In contrast, the expression of surface PD-1 in *Rab37* KO tumor-infiltrating CD8 T cells was reduced compared to that in *Rab37* WT tumor-infiltrating CD8 T cells (Fig. 4C). Furthermore, the level of exhaustion markers (PD-1 and TIM3) was significantly diminished in *Rab37* KO tumor-infiltrating CD8^+^ T cells compared to that in *Rab37* WT tumor-infiltrating CD8^+^ T cells (Fig. 4C). Moreover, the results of IF-IHC assay indicated that the colocalization levels of Rab37, PD-1 and LAG3 were increased in tumor infiltrating CD8^+^ T cells in *Rab37* WT group (Fig. 4D, F). However, tumor-infiltrating CD8^+^ T cells obviously reduced the expression of exhaustion markers (PD-1 and LAG3) in *Rab37* KO tumors (Fig. 4E, F). These in vivo results indicated that Rab37 suppressed effector CD8 T cells, thereby promoting tumor growth in lung cancer.

The mixed lymphocyte tumor cell culture (MLTC) has been conducted in attempts to generate lymphocytes with activities against tumor cells [22–24]. To clarify the impact of Rab37 on the functions of T cells, MLTC was used to investigate the function of splenocytes derived from LLC tumor-bearing *Rab37* WT and KO mice under anti-CD3/CD28 antibodies treatment by flow cytometry in the mix-culture system with LLC cancer cells (Additional file 1: Fig. S4A). We found that Ki67 was upregulated in *Rab37* KO splenic T cells compared to WT splenic T cells (Additional file 1: Fig. S4B). CD107a is required for perforin delivery to lytic granules for degranulation in activated CD8 T cells [25]. CD107a expression was increased in *Rab37* KO splenic T cells compared to WT splenic T cells (Additional file 1: Fig. S4C). Next, we determined whether Rab37-mediated T cell exhaustion characterized by the co-expression of PD-1 and another cell surface inhibitory molecule, TIM3. Flow cytometry results demonstrated that the PM presentation of PD-1^+^TIM3^+^ were downregulated in *Rab37* KO splenic CD8^+^ T cells compared to WT splenic CD8^+^ T cells (Additional file 1: Fig. S4D). These results show that T cell proliferation and cytolytic activity are relatively enhanced in *Rab37* KO-derived splenic T cells under MLTC condition.

To further confirm the effect of *Rab37* KO on the activity of CD8^+^ T cells, splenic CD8^+^ T cells isolated from *Rab37* KO or WT mice were subcutaneously co-injected with LLC cells into the *Rag1*-deficient mice with no mature T lymphocytes (Additional file 1: Fig. S4E). The tumor weight and volume measurements showed that *Rab37* KO CD8^+^ T cells suppressed the LLC tumor growth in the *Rag1*-deficient mice (Additional file 1: Fig. S4F–H). Notably, flow cytometry results showed that PD-1^+^ membrane presentation level was lower in *Rab37* KO splenic CD8^+^ T cells than those of WT splenic CD8^+^ T cells stimulated by anti-CD3/CD28 antibodies for 48 h (Additional file 1: Fig. S4I). These data suggest that Rab37 deficiency increases the anti-tumor activity of CD8^+^ T cells via reduction of PD-1 membrane presentation.

Effector CD4 T cells have multifaceted role in supporting maintenance of functional CD8 T cells [26]. Therefore, MLTC was used to examine the proliferation of splenic CD4^+^ T cells derived from tumor-bearing *Rab37* WT and KO mice by CFSE assay (Additional file 1: Fig. S4J). The flow cytometry data showed that the percentage of CFSE-FITC fluorescence was increased in *Rab37* KO splenic CD4^+^ T cells compared with *Rab37* WT group, suggesting that Rab37 deficiency increases the proliferation of CD4^+^ T cells via reduction of PD-1 membrane presentation (Additional file 1: Fig. S4K). These results illustrate that Rab37-mediated PD-1 sustainable PM presentation inhibits the functionality in both CD4^+^ and CD8^+^ T cells.

To further verify the significance of Rab37-mediated PD-1 PM presentation in PBMCs from nine lung cancer patients, we cultured human PBMCs for 7 days and then analyzed *Rab37* mRNA expression, PD-1^+^/TIM3^+^/CD8^+^ expression, GzmB^+^/IFN-γ^+^/CD8^+^ expression and cancer cell viability of the PBMCs (Fig. 4G). The results showed that PBMCs from lung cancer patients exhibited significant positive correlation between *Rab37* expression and population of PD-1^+^/TIM3^+^/CD8^+^ T cells (Fig. 4H), which accordingly had negative correlation with anti-tumor activity (GzmB^+^/IFN-γ^+^) in CD8^+^ T cells (Fig. 4I). The percentage of cancer cell viability was measured in PBMCs from three healthy donors and nine lung cancer patients (Fig. 4J). These effector T cells from PBMCs treated with CD3 and CD28 antibodies could secrete IFN-γ and TNF-α to act as cytotoxic cytokines together with granzyme B and perforin to initiate apoptosis in tumor cells, thereby killing cancer cells [27, 28]. In contrast, PBMCs derived from lung patients had reduced tumoricidal ability (Fig. 4J). Taken together, these results confirm that increased PD-1 PM presentation mediated by Rab37 overexpression inhibits proliferation and activities of T cells, thereby leading to T cell dysfunction.

### Rab37-expressing stroma cells correlated with the exhausted status of PD-1^+^/TIM3^+^ level in infiltrating CD8^+^ T cells in tumor specimens from lung cancer patients

Although CD8^+^ T cells are beneficial for tumor killing in tumor-infiltrating lymphocytes (TILs), these cells are often dysfunctional and effector subsets are reduced during tumor progression [2]. Based on our finding that overexpression of Rab37 mediated sustainable PD-1 PM presentation and T cell exhaustion, we thus proposed that the TIL populations of Rab37^+^/PD-1^+^/TIM3^+^/CD8^+^ T cells may be related to the adverse clinical responses in lung cancer patients.

**Fig. 5:**
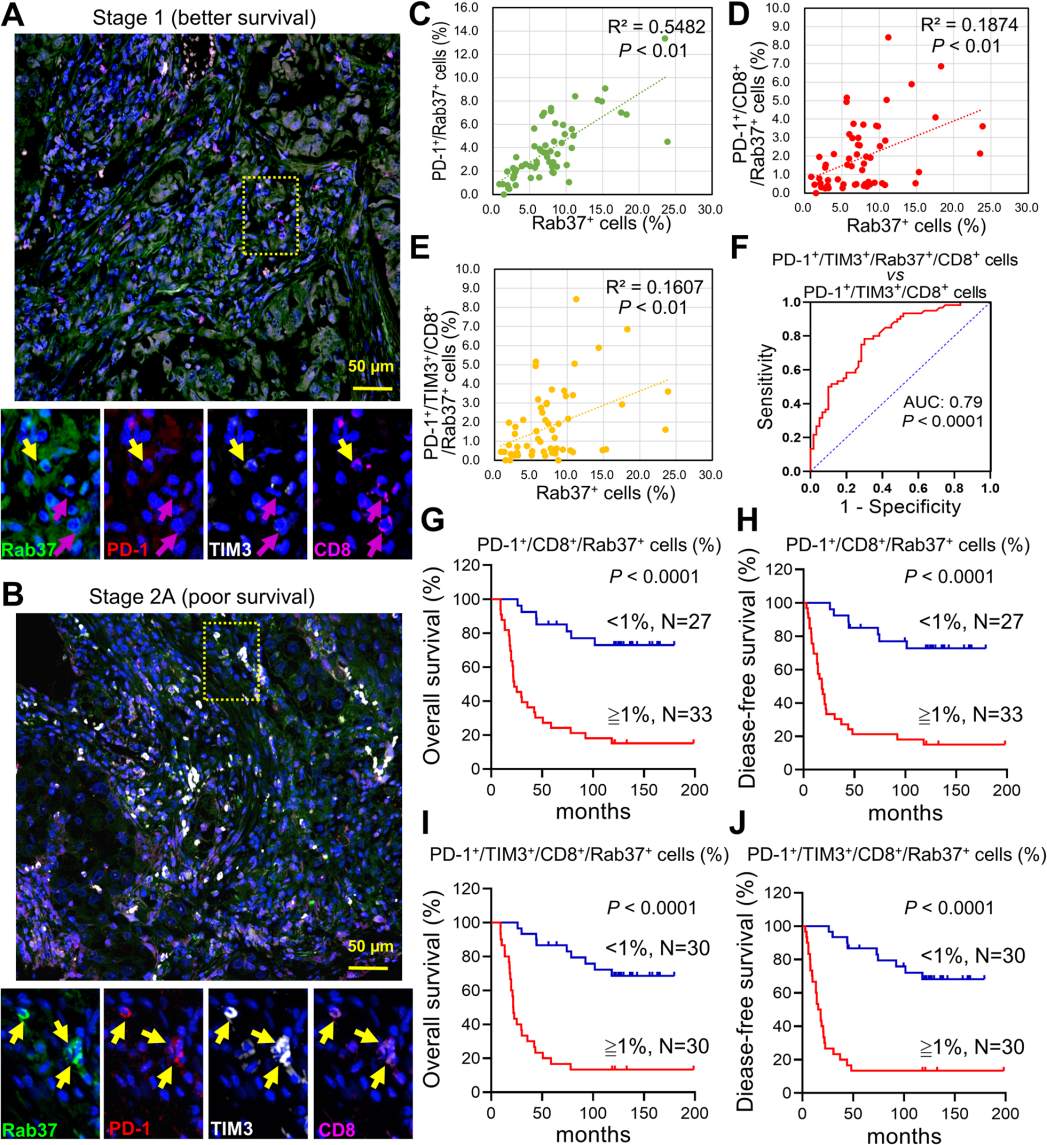
PD-1^+^/TIM3^+^/CD8^+^/Rab37^+^ cells serve as a prognostic biomarker in lung cancer. **A**, **B** Paraffin wax-embedded tissue sections of tumor derived from early-stage lung cancer patients with good survival (**A**) or with poor survival (**B**) are shown. Corresponding region of interests (ROIs) were captured for individual analysis based on IF staining for Rab37 (green), PD-1 (red), TIM3 (white) or CD8 (purple). Blue signals represents DAPI (nucleus staining). Enlarged panels of the inset in the merged image are shown below. Colocalization of Rab37, PD-1 and TIM3 in CD8^+^ T cells were detected by merging the mono-staining pictures. Yellow arrows are CD8^+^ T cells expressing Rab37, PD-1 and TIM3 (exhausted CD8^+^ T cells), while purple arrows are CD8^+^ T cells without expressing Rab37, PD-1 and TIM3 (activated CD8^+^ T cells). Scale bar: 50 μm. **C**–**E** Scatter plot showing the correlation between Rab37^+^ cells and stromal PD-1^+^/Rab37^+^ cells (**C**), PD-1^+^/CD8^+^/Rab37^+^ cells (**D**), and PD-1^+^/TIM3^+^/CD8^+^/Rab37^+^ cells (**E**) in lung cancer patients. Pearson correlation coefficient, R square and P value are shown. **F** ROC curve of PD-1^+^/TIM3^+^/CD8^+^ *vs* PD-1^+^/Rab37^+^/TIM3^+^/CD8^+^ cells suggested that the tumor-infiltrating PD-1^+^/Rab37^+^/TIM3^+^/CD8^+^ T cells as a potential biomarker with improved AUC. **G**–**J** Kaplan–Meier survival analysis demonstrated that lung cancer patients with high PD-1^+^/CD8^+^/Rab37^+^ or PD-1^+^/TIM3^+^/CD8^+^/Rab37^+^ cells expression showed the worst overall survival (**G**, **I**) and disease-free survival (**H**, **J**). Patients with PD-1^+^/CD8^+^/Rab37^+^ or PD-1^+^/TIM3^+^/CD8^+^/Rab37^+^ cells ≥ 1% was defined as high expression. P-values were determined using log-rank test

To this end, we examined TILs on surgical tumor specimens of 60 lung cancer patients. Figure 5A, B illustrates tumor sections from two patients at the early stage of disease, but one patient had better survival than the other. The overall colocalization levels of Rab37, PD-1 and TIM3 were low in tumor-infiltrating CD8^+^ T cells in patients with better survival (Fig. 5A), whereas the colocalization levels of Rab37^+^/PD-1^+^/TIM3^+^/CD8^+^ T cells were increased in the patients with poor survival (Fig. 5B). The quantitative analysis indicated that the regions enriched in PD-1^+^/Rab37^+^ cells (R square = 0.5482, *P* < 0.01, n = 60) (Fig. 5C), PD-1^+^/CD8^+^/Rab37^+^ T cells (R square = 0.1874, *P* < 0.01, n = 60) (Fig. 5D), and PD-1^+^/TIM3^+^/CD8^+^/Rab37^+^ T cells (R square = 0.1607, *P* < 0.01, n = 60) (Fig. 5E) positively correlated with high Rab37^+^ stains. Moreover, ROC method was performed on PD-1^+^/TIM3^+^/CD8^+^ T cells and PD-1^+^/TIM3^+^/CD8^+^/Rab37^+^ T cells. The results suggested that tumor-infiltrating PD-1^+^/TIM3^+^/CD8^+^/Rab37^+^ T cells served as a potential biomarker for lung cancer prognosis (AUC: 0.79, *P* < 0.0001) (Fig. 5F).

Next, the Kaplan–Meier analysis results showed that increased intratumoral stain intensity of PD-1^+^/CD8^+^/Rab37^+^ and PD-1^+^/TIM3^+^/CD8^+^/Rab37^+^ T cells correlated with poorer overall survival and disease-free survival of lung cancer patients (Fig. 5G–J). These clinical data suggest that tumors from lung cancer patients with poor survival are characterized by a distinct immunosuppressive TME with a high level of tumor-infiltrating Rab37^+^/PD-1^+^/TIM3^+^/CD8^+^ T cells.

Moreover, we performed correlation analysis of intratumoral stain intensity of Rab37^+^/PD-1^+^/TIM3^+^/CD8^+^ T cells with clinicopathological parameters of lung cancer. The results showed that patients with high tumor-infiltrating Rab37^+^/PD-1^+^/TIM3^+^/CD8^+^ T cell signals correlated with cancer recurrence (Table 1). Moreover, we performed univariate and multivariate Cox regression analyses in this cohorts of lung cancer patients (Table 2). Univariate Cox regression analysis indicated that lung cancer patients with high tumor-infiltrating Rab37^+^/PD-1^+^/TIM3^+^/CD8^+^ T cell profile or aggressive M status showed poor survival outcome. Importantly, multivariate Cox regression analysis revealed that lung cancer patients with high tumor-infiltrating Rab37^+^/PD-1^+^/TIM3^+^/CD8^+^ T cell profile (hazard ratio = 6.074, *P* = 0.001) still showed significantly high risk of death even after adjusting for other clinical parameters (Table 2). Taken together, these results indicate that high tumor-infiltrating Rab37^+^/PD-1^+^/TIM3^+^/CD8^+^ T cell level could be used as an independent factor to predict clinical outcome in patients with lung cancer.

**Table 1:** Alteration of Rab37^+^/PD-1^+^/TIM3^+^/CD8^+^ cells in relation to clinicopathological parameters in tumor specimen from 60 lung cancer patients

Clinical parameters	Total Rab37^+^/PD-1^+^/TIM3^+^/CD8^+^ cells (%)			*P*-value^a^
	Patients	Protein expression		
	60	N = 30 (50%) Low expression^b^	N = 30 (50%) High expression^b^	
Age				
< 65	36	15 (41.7)	21 (58.3)	0.187
≥ 65	24	15 (62.5)	9 (37.5)	
Gender				
Female	37	21 (56.7)	16 (43.2)	0.288
Male	23	9 (39.1)	14 (61.8)	
Tumor type				
ADC	46	21(48.8)	25(51.1)	0.360
SCC	14	9(64.2)	5(35.7)	
Tumor stage				
I–II	36	22 (61.1)	14 (38.8)	0.064
III–IV	24	8 (33.3)	16 (66.6)	
T stage^c^				
T1	18	12 (66.6)	6 (33.3)	0.262
T2	28	12 (42.8)	16 (57.1)	
T3	8	3 (37.5)	5 (62.5)	
T4	6	3 (50.0)	3 (50.0)	
N stage^c^				
≤ N1	22	16 (72.7)	6 (27.7)	**0.015**
> N1	38	14 (36.8)	24 (63.1)	
M stage^c^				
M0	55	30 (54.5)	25 (45.4)	0.052
≥ M1	5	0 (0.0)	5 (100.0)	
Differentiation grade				
Well	7	3 (42.8)	4 (57.1)	0.227
Moderate	40	23 (57.5)	17 (42.5)	
Poor	13	4 (30.7)	9 (69.2)	
Recurrence				
No	19	16 (84.2)	3 (15.7)	**0.001**
Yes	41	14 (34.1)	27 (65.8)	

*ADC* adenocarcinoma, *SCC* squamous cell carcinoma

^a^The data were analyzed by Pearson χ^2^ test. P values with statistical significance are in bold font (*P* < 0.05)

^b^Patient with Rab37^+^/PD-1^+^/TIM3^+^/CD8^+^ cells (%) ≥ 1% was defined as high expression

^c^T stage: primary tumor; N stage: lymph node metastasis; M stage: distant metastasis

**Table 2:** Cox regression analysis of risk factors for cancer-related death in lung cancer patients

Characteristics	Univariate analysis		Multivariate analysis	
	HR (95% CI)	*P*-value^a^	HR (95% CI)	*P*-value^a^
Rab37^+/^PD-1^+^/TIM3^+^/CD8^+^ cells (%)^b^				
< 1%	1		1	
≥ 1%	7.089 (3.251–15.459)	**0.001**	6.074 (2.715–13.588)	**0.001**
Age				
< 65	1		–^d^	
≥ 65	0.724 (0.364–1.440)	0.351	–^d^	–^d^
Gender				
Female	1		–^d^	
Male	1.839 (0.944–3.583)	0.078	–^d^	–^d^
Tumor type				
ADC	1		–^d^	
SCC	1.409 (0.614–3.235)	0.403	–^d^	–^d^
Stage				
I–II	1		1	
III–IV	2.769 (1.407–5.449)	**0.003**	1.855 (0.905–3.927)	0.091
T stage^c^				
T1	1		–^d^	
T2	1.322 (0.589–2.967)	0.499	–^d^	–^d^
T3	2.073 (0.733–5.863)	0.17	–^d^	–^d^
T4	1.103 (0.298–4.082)	0.884	–^d^	–^d^
N stage^c^				
≤ N1	1		–^d^	
> N1	1.913 (0.916–3.996)	0.084	–^d^	–^d^
M stage^c^				
M0	1		1	
≥ M1	4.638 (1.709–12.591)	**0.003**	1.498 (0.519–4.326)	0.455

*HR* hazard ratio, *CI* confidence interval, *ADC* adenocarcinoma, *SCC* squamous cell carcinoma

^a^P values with statistical significance are in bold font (*P* < 0.05)

^b^Patient with Rab37^+^/PD-1^+^/TIM3^+^/CD8^+^ cells (%) ≥ 1% was defined as high expression

^c^T status: primary tumor; N status: lymph node metastasis; M status: distant metastasis

^d^The variables without significant HR in the univariate analysis were not included in the multivariate analysis

## Discussion

**Fig. 6:**
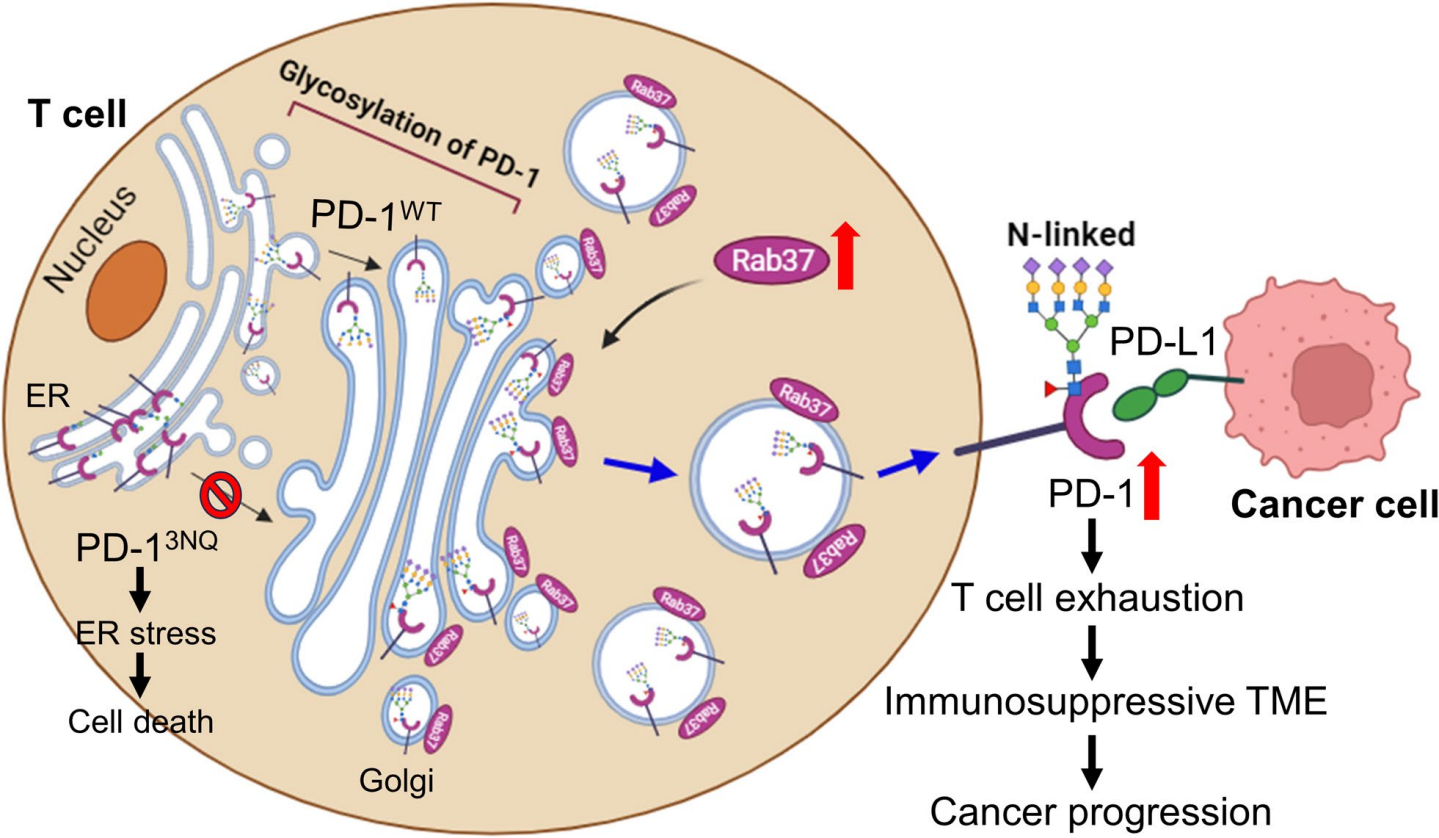
Schematic diagram of Rab37-mediated intracellular trafficking and plasma membrane presentation of PD-1 to sustain T cell exhaustion and tumor progression. Rab37, a membrane attached GTPase, is upregulated in tumor-infiltrating T cell to mediate the membrane transport of PD-1 via the Rab37-specific vesicles. Proper PD-1 glycosylation is required for Rab37-mediated vesicular transport between the Golgi apparatus and the plasma membrane. Rab37 sustains surface PD-1 and leads to T cell exhaustion to foster an immunosuppressive TME and cancer progression (right). In contrast, glycol-mutant PD-1^3NQ^ proteins largely accumulate at ER site resulting in ER stress and leading to cell death (left)

Our study reveals a novel regulatory mechanism of Rab37-mediated PD-1 membrane trafficking that leads to T cell exhaustion (Fig. 6). We disclosed that upregulation of Rab37 facilitates PD-1 PM presentation in T cells. Sustained surface PD-1 leads to suppression of proliferation and activation in T cells, thereby causing T cell exhaustion and fostering an immunosuppressive TME. Proper PD-1 glycosylation is required for Rab37-mediated vesicular transport between the Golgi apparatus and the PM. In addition, we identify that the increased infiltration of Rab37^+^/PD-1^+^/TIM3^+^ T cells in tumor serves as a biomarker of poor prognosis in lung cancer patients.

To date, the knowledge of how PD-1 is being trafficked to the PM remains quite limited. Delivery of the PD-1 into the immunological synapse is essential for equilibrium in T cell activation, whereas upregulation of surface PD-1 leads to loss of T cell effector function in the presence of PD-L1 in TME [7, 29]. Our results reveal for the first time that Rab37 mediates PD-1 intracellular trafficking and PM presentation to sustain T cell exhaustion in lung cancer. Notably, Rab37 expression level was increased in Jurkat T cells upon lung cancer CM treatment (Fig. 1H). The tumor regions enriched in PD-1^+^/TIM3^+^/CD8^+^ T cells also positively correlated with high Rab37^+^ stains in lung cancer patients (Fig. 5). Similarly, human PBMCs from lung cancer patients with high expression of Rab37 had positive correlation with the population of PD-1^+^/TIM3^+^/CD8^+^ T cell (Fig. 4H). Notably, from lung cancer patients showed higher level of *Rab37* expression than PBMCs from healthy donors (Additional file 1: Fig. S6), consistent with our in vitro observation that Rab37 was upregulated in T cells upon cancer CM treatment (Fig. 1H). The paracrine factors or signals for upregulation of Rab37, and subsequent increased presentation of membrane PD-1 in the TME are yet to be determined. Nevertheless, Rab37 expression can be regulated through transcriptional control. Xu et al. identified some transcriptional factors such as E2F1 that drive *Rab37* gene expression [30]. We previously reported that *Rab37* is hypermethylated in lung cancer, thereby leading to downregulation of Rab37 [31]. In the TME, epigenetic regulation plays an important role in the turn-on or turn-off of gene upon external factors stimulation. The detailed mechanism of switching Rab37 expression in the TME is worth future investigation.

Several known Rab and SNARE proteins are involved in trafficking processes of signaling molecules and cytokines at the immunological synapse in response to T cell receptor (TCR) activation. For example, Rab3d and Rab8b deliver the newly synthesized TCR to the immunological synapse in Jurkat T cells [32], while Rab35 involvement in TCR recycling has been confirmed in Th2 cells [33]. In addition, exocytic Rab8, Rab27a, and Rab37 work together to regulate lytic granule and cytokine secretion [32, 34, 35]. Notably, our findings excluded the involvement of Rab3, Rab8, and Rab11 for PD-1 PM presentation in tumor-specific T cells. As regard to the target membrane SNARE proteins (tSNARE), SNAP23 and syntaxin-4 colocalize with TCR at the immunological synapse upon TCR activation in Jurkat cells [36]. In addition, vesicle SNARE proteins (vSNARE), VAMP2, VAMP3, VAMP7 and tSNARE Vti1B have been demonstrated to mediate TCR complex trafficking [37, 38]. It is worthy to investigate whether these Rab proteins and SNAREs could also be involved in Rab37-mediated PD-1 vesicle trafficking to the PM in tumor-specific T cells.

Notably, N-linked glycosylation is required for PD-1 recruitment and membrane trafficking. N-glycan mutation on extracellular domain in gPD-1 causes a delay in intracellular vesicle trafficking and eventually cell death. N-glycans are often added to the membrane protein to aid in the folding process and usually enhance protein stability. Indeed, the GFP-3NQ-gPD-1 group was less stable and easily degraded compared with GFP-WT-gPD-1 group (Additional file 1: Fig. S3H, I). These results explain the reduced gPD-1 expression on the PM in GFP-3NQ-gPD-1 group. However, we could not exclude the possibility that other glycosylation modifications on PD-1 may be required. Sun and her colleagues found that PD-1-specific glycoforms including N-glycosylation, *N*-acetyl-glucosamine and sialic acid are important in TCR activation [13]. The underlying mechanism of additional glycosylation modifications on PD-1 for membrane trafficking and presentation warrants further investigation.

Although ICB therapy is promising for achieving long-term efficacy, most patients who initially responded to treatment inevitably developed drug resistance to ICB therapy after a period of treatment. The mechanisms of tumor immune resistance are very complex and involve multiple aspects such as genes, metabolism, inflammation, abnormal neovascularization, the changes of immunosuppressive cells, immunosuppressive cytokines, coinhibitory receptors, and costimulatory receptors in the TME. Moreover, immunosuppressive TME often contain exosomal PD-L1, immunosuppressive cells such as myeloid-derived suppressor cells, regulatory T cells, and tumor-associated macrophages which contribute to promote activation of coinhibitory receptors (e.g., PD-L1, CTLA-4, TIM3, TIGHT) and inhibition of costimulatory receptor (e.g., CD28). Ultimately, cytotoxic CD8 T cells gradually lose their anti-tumor function leading to ICB resistance in cancer patients [39].

ICB therapy mainly refers to improvement in exhausted CD8 T cell function. Exhaustion has been identified as a dynamic process from progenitor exhaustion through to terminally exhaustion in tumor development. Progenitor exhausted T cells are long-lived with stem-like properties. On the other hand, terminally exhausted T cells are short-lived, which is considered as a temporary effect in eliminating tumor cells [40, 41]. Reversing T cell exhaustion by blocking the PD-1/PD-L1 pathway is a common strategy to control tumor growth. High levels of surface PD-1 are regarded as an activator of terminally exhausted T cells [42]. Principally, reversing T cell exhaustion by immunotherapy indicates that exhausted T cells are not completely terminal. In other words, antibody blockade of PD-1/PD-L1 can enhance the cytotoxic ability of progenitor exhausted T cells with intermediate levels of PD-1 to suppress tumor growth [8]. Importantly, lung cancer patients with high infiltrating Rab37^+^/PD-1^+^/TIM3^+^ CD8^+^ T cells showed poor prognosis, suggesting that these tumor-infiltrating T cells are likely terminal exhaustion T cells with reduced proliferation and poor responses to immunotherapy. Targeting PD-1, CTLA4, LAG3, or TIM3 axis may be a potential immunotherapy-based combination strategy to enhance population of cytotoxic effector T cells in patients with high level of Rab37^+^/PD-1^+^/TIM3^+^ tumor-infiltrating CD8^+^ T cells [40–45].

Beyond the antibodies against inhibitory receptors, dendritic cell (DC)-based immunotherapy demonstrates remarkable clinical prospect through different biomaterials to optimize the function of DCs. These biomaterials may serve as antigen carriers to prolong antigenic exposure to DCs or deliver antigens via integration of specific ligands or antibodies on their cell surface, which in turn sustainably stimulate and activate TCRs on T cells [46–48]. On the other hand, endogenous/exogenous nanovaccine also can synergistically facilitate DC-mediated tumor immunotherapy. Nanovaccine can generate endogenous danger signals and antigen release to boost vaccination for DC maturation and antigen cross-presentation. In addition, exogenous nanovaccine can deliver model antigen ovalbumin and agonists (CpG-ODN) to further enhance DC activation for T cell activation [46–48].

In our study, upregulation of Rab37 in T cells by the TME results in sustainable PD-1 PM presentation accompanied with other co-inhibitory receptor TIM3 expression and reduced anti-tumor activity. In addition, our ex vivo results indicated that PBMCs from lung cancer patients with high expression of Rab37 positively correlated with the population of PD-1^+^/TIM3^+^CD8^+^ T cells accompanied with negative correlation with anti-tumor activity (GzmB^+^/IFN-γ^+^) in CD8^+^ T cells and diminished tumoricidal activity. Together, high Rab37 expression in T cells could be a potential biomarker to predict poor responses to ICB therapy.

## Conclusions

PD-1 plays an important role in tumor immunity; however, the molecular regulation of PD-1 transportation remains elusive. Herein, we uncover that Rab37 mediates glycosylated PD-1 trafficking to the PM in T cells, thereby facilitating T cell exhaustion. Lung cancer patients with high levels of intratumoral Rab37^+^PD-1^+^TIM3^+^CD8^+^ T cells are found to be significantly associated with poor prognosis. Human PBMCs from lung patients also exhibited high expression of Rab37, which positively correlated with elevated levels of PD-1^+^ and TIM3^+^ in CD8^+^ T cells. Our findings provide a novel molecular mechanism in Rab37-mediated regulation of PD-1 membrane presentation that reinforces T cell exhaustion, which could be a novel biomarker for ICB therapy.

### Supplementary Information

**Additional file 1: Table S1.** The plasmids and their characteristics used in the current study. **Table S2.** Antibodies and their reaction conditions used in the current study. **Table S3.** Characteristics of NSCLC patients and normal individuals for ex vivo assays in the current study. **Figure S1.** The expression of PD-1 and Rab37 exhibited positive correlation in T cells. **Figure S2.** Rab37 mediates PD-1 membrane trafficking. **Figure S3.** Glycosylation on PD-1 promotes protein stability and transport to the PM. **Figure S4.** Rab37-mediated PD-1 PM presentation reduces T cell function. **Figure S5.** The serum biochemical parameters and major organ histology in LLC tumor-bearing *Rab37* KO and WT mice. **Figure S6.** The relationship between Rab37 expression and population of PD-1/TIM3/CD8 cells derived from PBMCs treated with CD3 and CD28 antibodies.

**Additional file 2: Movie S1.** Time-lapse movie of TIRF images in 293T cells expressing GFP-PD-1 and RFP-tagged Rab37. Images were captured with TIRF microscope at 491 and 561 nm laser every 6 s over a period of 18 s. Time intervals in minutes and seconds are shown. Stills presenting in 00:01 to 00:04 of this movie correspond to 00:00 to 00:18 in Fig. 2F (RFP-Rab37/GFP-PD-1). Scale bars: 20 μm.

**Additional file 3: Movie S2.** Time-lapse movie of TIRF images in 293T cells expressing GFP-PD-1 with RFP-tagged EV. Images were captured with TIRF microscope at 491 and 561 nm laser every 10 s over a period of 30 s. Time intervals in minutes and seconds are shown. Stills presenting in 00:01 to 00:08 of this movie correspond to 00:00 to 00:30 in Fig. 2G (RFP-EV/GFP-PD-1). Scale bars: 20 μm.

**Additional file 4: Movie S3.** Time-lapse movie of confocal images in 293T cells expressing GFP-PD-1 and RFP-tagged Rab37. Images were captured with live confocal fluorescence microscope at 491 and 561 nm laser every 10 s over a period of 50 s. Time intervals in minutes and seconds are shown in Figure S2C (RFP-Rab37/GFP-PD-1). Scale bars: 10 μm.

**Additional file 5: Movie S4.** Time-lapse movie of confocal images in 293T cells expressing GFP-PD-1 with RFP-tagged EV. Images were captured with live confocal fluorescence microscope at 491 and 561 nm laser every 10 s over a period of 50 s. Time intervals in minutes and seconds are shown in Figure S2D (RFP-EV/GFP-PD-1). Scale bars: 10 μm.

## Data Availability

All data used during the current study available from the corresponding author on reasonable request.

## Abbreviations

AUC: Area under the ROC curve
CM: Conditioned medium
CI: Confidence interval
DC: Dendritic cell
EV: Empty vector
gPD-1: Glycosylation of PD-1
GEPIA: Gene expression profiling interactive analysis
GFP-PD-1: Green fluorescent protein-tagged PD-1
GzmB: Granzyme B
ICB: Immune checkpoint blockade
Io: Ionomycin
IF-IHC: Immunofluorescent immunohistochemistry
IP: Immunoprecipitation
IF: Immunofluorescence
ITSM: Immunoreceptor tyrosine-based switch motif
ITIM: Immunoreceptor tyrosine-based inhibitory motif
KO: Knockout
LLC: Lewis lung carcinoma
MLTC: Mixed lymphocyte tumor cell culture
NFAT: Nuclear factor of activated T-cells
OE: Overexpressed
PBMC: Peripheral blood mononuclear cell
PD-1: Programmed cell death protein 1
PD-L1/2: Program cell death ligand 1/2
PM: Plasma membrane
PMA: Phorbol 12-myristate 13-acetate
RFP-Rab37: Red fluorescent protein-tagged Rab37
ROC: Receiver operating characteristic
SHPs: Src homology region 2 domain-containing phosphatases
Tex: Exhaustion T cell
TME: Tumor microenvironment
TCR: T cell receptor
TIRF: Total internal reflection fluorescence
TILs: Tumor-infiltrating lymphocytes
WT: Wild-type
