# Supplementary material for: Rab37 mediates trafficking and membrane presentation of PD-1 to sustain T cell exhaustion in lung cancer
Source: J Biomed Sci. 2024 Feb 7;31:20. doi: 10.1186/s12929-024-01009-6 (PMC10848371; doi:10.1186/s12929-024-01009-6)
Supplement: Supplementary file 1 — Additional file 1: Table S1. The plasmids and their characteristics used in the current study. Table S2. Antibodies and their reaction conditions used in the current study. Table S3. Characteristics of NSCLC patients and normal individuals for ex vivo assays in the current study. Figure S1. The expression of PD-1 and Rab37 exhibited positive correlation in T cells. Figure S2. Rab37 mediates PD-1 membrane trafficking. Figure S3. Glycosylation on PD-1 promotes protein stability and transport to the PM. Figure S4. Rab37-mediated PD-1 PM presentation reduces T cell function. Figure S5. The serum biochemical parameters and major organ histology in LLC tumor-bearing Rab37 KO and WT mice. Figure S6. The relationship between Rab37 expression and population of PD-1/TIM3/CD8 cells derived from PBMCs treated with CD3 and CD28 antibodies. [file 12929_2024_1009_MOESM1_ESM.pdf]

Rab37 mediates trafficking and membrane presentation of PD-1 to sustain T cell exhaustion in lung cancer

Wan-Ting Kuo, I-Ying Kuo, Hung Chia Hsieh, Ssu-Ting Wu, Wu-Chou Su, and Yi-Ching Wang

Supplemental Data

Supplementary Table 1

Supplementary Table 2

Supplementary Table 3

Supplementary Figure 1

Supplementary Figure 2

Supplementary Figure 3

Supplementary Figure 4

Supplementary Figure 5

Supplementary Figure 6

Supplementary Movie 1

Supplementary Movie 2

Supplementary Movie 3

Supplementary Movie 4

Table S1. The plasmids and their characteristics used in the current study.

Plasmid	Target	Insert (bp)	Function	Source
pcDNA3.1-V5/His-vector	None	--[a]	Vector control	Invitrogen
pCMV3- hRab37-WT-OFPSpark	Wild type human Rab37	672	Overexpression	Homemade[b]
pcDNA3.1-V5/His-hRab37-WT	Wild type human Rab37	672	Overexpression	Homemade[b]
pcDNA3.1-V5/His-hRab37-QL	Q89L human Rab37	672	Overexpression	Homemade[b]
pcDNA3.1-V5/His-hRab37-TN	T43N human Rab37	672	Overexpression	Homemade[b]
pcDNA3.1-V5/His-hPD-1-GFP	Wild type human PD-1	867	Overexpression	Sino Biological Inc
pcDNA3.1-V5/His -N49Q-PD-1-GFP	N49Q human PD-1	867	Overexpression	Homemade[c]
pcDNA3.1-V5/His -N58Q-PD-1-GFP	N58Q human PD-1	867	Overexpression	Homemade[c]
pcDNA3.1-V5/His -N74Q-PD-1-GFP	N74Q human PD-1	867	Overexpression	Homemade[c]
pcDNA3.1-V5/His-N116Q-PD-1-GFP	N116Q human PD-1	867	Overexpression	Homemade[c]
pcDNA3.1-V5/His -2NQ-PD-1-GFP	N49/58Q human PD-1	867	Overexpression	Homemade[c]
pcDNA3.1-V5/His -3NQ-PD-1-GFP	N49/58/74Q human PD-1	867	Overexpression	Homemade[c]
pGL4.30[luc2P/NFAT-RE/Hygro]	NFAT response element	90	Reporter vector	Promega

[a] The plasmid is used as a backbone vector therefore there is no inserted DNA fragment.

[b] Rab37-WT, Q89L or T43N was PCR-amplified with designated mutation at the primer sequences and cloned into pcDNA3.1-V5/His or pCMV3-OFPSpark expression vector to generate V5/His-tagged or RFP-tagged Rab37 expression vector.

[c] PD-1-WT, N49Q, N58Q, N74Q, N116Q, 2NQ, 3NQ, were PCR-amplified with designated mutation at the primer sequences and cloned into pcDNA3.1-V5/His expression vector to generate GFP-tagged PD-1 expression vector.

Table S2. Antibodies and their reaction conditions used in the current study.

Target	KD	Raised in	Application	Dilution	Source	Catalog no.
Rab37	27	Rabbit	Western blot	1:1000	Proteintech	13051-1-AP
			Immunofluorescence	1:5000		
	--[a]	Mouse	Immuno-EM	1:30	Homemade	
PD-1	32	Rabbit	Western blot	1:1000	Genetex	GTX31309
	--[a]	Hamster	Flow cytometry (APC)	1:200	BD Bioscience	562671
	--[a]	Mouse	Flow cytometry (BB515)	1:200	BD Bioscience	564494
PD-1	55	Rabbit	Western blot	1:1000	Cell Signaling Technology	86163
	--[a]		Immuno-EM	1:20		
			Immunofluorescence	1:2000		
V5 tag	--[a]	Mouse	Western blot	1:5000	Invitrogen	46-0705
Calnexin	68	Rabbit	Western blot	1:5000	Genetex	GTX109669
β-ACTIN	42	Mouse	Western blot	1:5000	Genetex	GTX26276
PERK	125	Rabbit	Western blot	1:1000	Genetex	GTX129275
Phospho PERK	125	Rabbit	Western blot	1:1000	Cell Signaling	3179S
eIF2α	38	Rabbit	Western blot	1:1000	Genetex	GTX101241
Phospho eIF2α	38	Rabbit	Western blot	1:1000	Cell Signaling	3398S

ATF6	110, 65	Rabbit	Western blot	1:1000	Genetex	GTX104820
XBP1s	50, 30	Rabbit	Western blot	1:1000	Cell Signaling	12782S
Rab11b	25	Rabbit	Western blot	1:1000	Genetex	GTX119095
			Immunofluorescence	1:200		
Rab8a	25	Rabbit	Western blot	1:1000	elabscience	EC8703
Rab3a	25	Rabbit	Western blot	1:1000	Proteintech	15029-1-AP
GAPDH	37	Mouse	Western blot	1:1000	Santa Cruz	Sc-32233
Na^+/K^+ATPase	113	Rabbit	Western blot	1:100000	Abcam	ab76020
EEA1	--[a]	Rabbit	Immunofluorescence	1:200	Genetex	GTX109638
TGN46	--[a]	Mouse	Immunofluorescence	1:200	Genetex	GTX22809
CD3	--[a]	Mouse	Cell culture	1:1000	BD Bioscience	566685
CD28	--[a]	Mouse	Cell culture	1:1000	BD Bioscience	555725
CD4	--[a]	Rat	Flow cytometry (FITC)	1:200	BD Bioscience	553047
CD8	--[a]	Rat	Flow cytometry (FITC)	1:200	BD Bioscience	553031
		Mouse	Flow cytometry (APC)	1:200	BD Bioscience	561952
		Rabbit	Immunofluorescence	1:3000	Abcam	ab217344
TIM3	--[a]	Rabbit	Immunofluorescence	1:2000	Cell Signaling	45208T

	--[a]	Human	Flow cytometry (BV421)	1:200	BD Bioscience	565562
Ki67	--[a]	Rat	Flow cytometry (FITC)	1:200	eBioscience	2040334
		Mouse	Flow cytometry (BV510)	1:200	BD Bioscience	563462
CD107a/LAMP1	--[a]	Mouse	Flow cytometry (PE-Cy7)	1:200	BD Bioscience	561348
	--[a]	Rat	Flow cytometry (PerCP/Cy5.5)	1:200	BD Bioscience	121626
Granzyme B	--[a]	Mouse	Flow cytometry (PE)	1:200	BioLegend	372207
TNF-α	--[a]	Rat	Flow cytometry (BV421)	1:200	BD Bioscience	563387
CD25	--[a]	Rat	Flow cytometry (APC)	1:200	BD Bioscience	557192
DAPI	--[a]	-[c]	Immunofluorescence	-[b]	Genetex	GTX30920
Opal 520 Reagent	--[a]	-[c]	Florescent immunohistochemistry	1:100	Akoya Biosciences	FP1013
Opal 570 Reagent	--[a]	-[c]	Florescent immunohistochemistry	1:100	Akoya Biosciences	FP1014
Opal 620 Reagent	--[a]	-[c]	Florescent immunohistochemistry	1:100	Akoya Biosciences	FP1495A
Opal 670 Reagent	--[a]	-[c]	Florescent immunohistochemistry	1:100	Akoya Biosciences	FP1117

[a] Molecular weight is not applicable to this antibody in such an application.

[b] DAPI is a commercial product for nuclear staining.

[c] Species is not applicable to this antibody in such an application.

Table S3. Characteristics of NSCLC patients and normal individuals for *ex vivo* assays in the current study.

**Clinical features**	**NSCLC patients**<br>**N = 9**	**Normal individuals**<br>**N = 3**
**Age: Median (range)**	71 (46-83)	29 (28-30)
**Sex**		
Male	8	3
Female	1	0
**Histology**		
Adenocarcinoma	6	- [a]
Squamous cell carcinoma	2	
Others	1	
***EGFR* gene status**		
Mutated	1	- [a]
Wild-type	7	
No data	1	
**Stage**		
I-III	0	- [a]
IV	9	

[a] Not applicable.

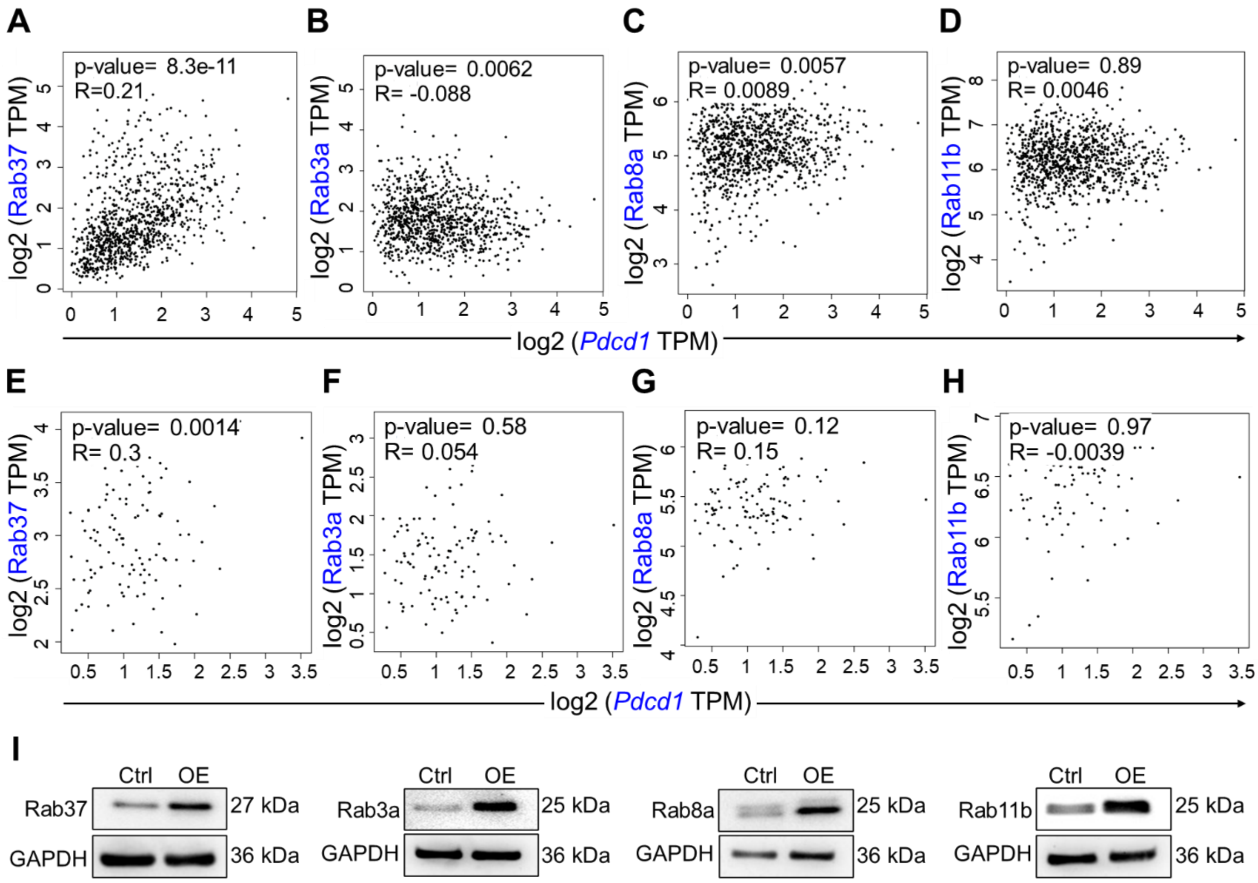

Figure S1. **The expression of PD-1 and Rab37 exhibited positive correlation in T cells.** The correlation of *Pdcd1* mRNA expression with Rab37, Rab3a, Rab8a or Rab11b in patients with lung adenocarcinoma (n=483) and lung squamous cell carcinoma (n=486) **(A-D)** or in normal tissues (n=109) **(E-H)** from the GEPIA dataset. R square and P value by Pearson correlation coefficient are shown. **(I)** Jurkat T cells were transfected with Rab37, Rab3a, Rab8a, or Rab11b plasmid for 24h, respectively, and protein expression were detected by immunoblotting. OE: overexpression.

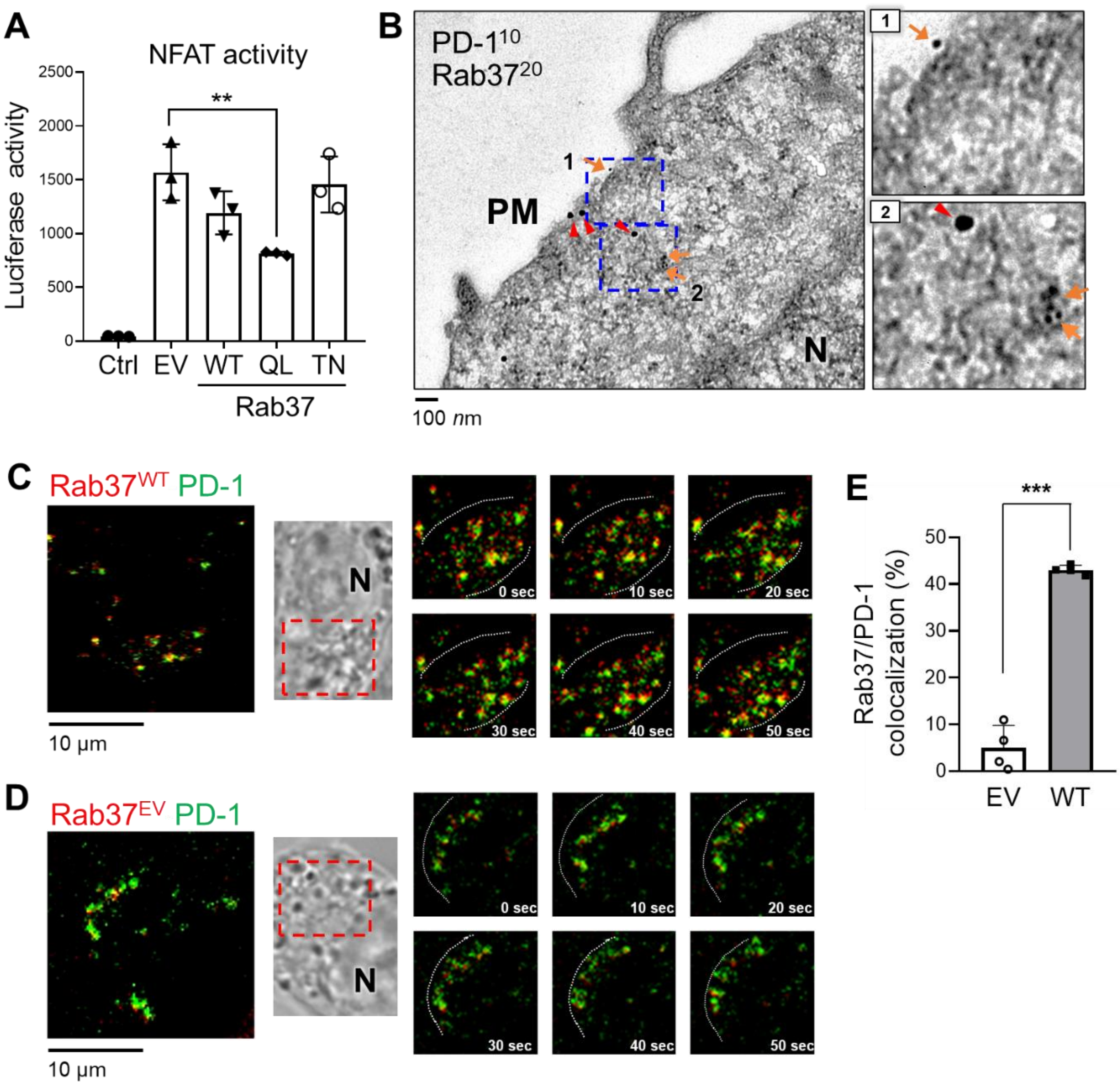

Figure S2. **Rab37 mediates PD-1 membrane trafficking. (A)** The NFAT activity was determined by luciferase reporter assay in Jurkat T cells overexpressing EV, Rab37-WT, Rab37-Q89L, or Rab37-T43N. **(B)** Ultrastructural localization of Rab37 (20 nm of gold, red triangle) and PD-1 (10 nm of gold, orange arrow) illustrated by immune-EM images. PM: plasma membrane, N: nucleus. Scale bars: 100 *n*m. **(C-D)** The dynamics of Rab37-regulated PD-1 trafficking by real-time confocal live images. Selected frames from time-lapse images Jurkat cells expressing RFP-Rab37 and GFP-PD-1 (C) or GFP-PD-1 with RFP-EV (D). Enlarged images of the boxed areas from **movies S3-S4** with time intervals in seconds are shown. Colocalized RFP-Rab37/GFP-PD-1 puncta are shown in yellow. Scale bar: 10 μm. **(E)** Quantification data of the colocalization between GFP-PD-1 and RFP-Rab37-WT in the intracellular cytoplasm. The data are shown as the mean ± S.D. P values were determined by two-tailed Student's t test. * P<0.05, ** P<0.01, *** P<0.001.

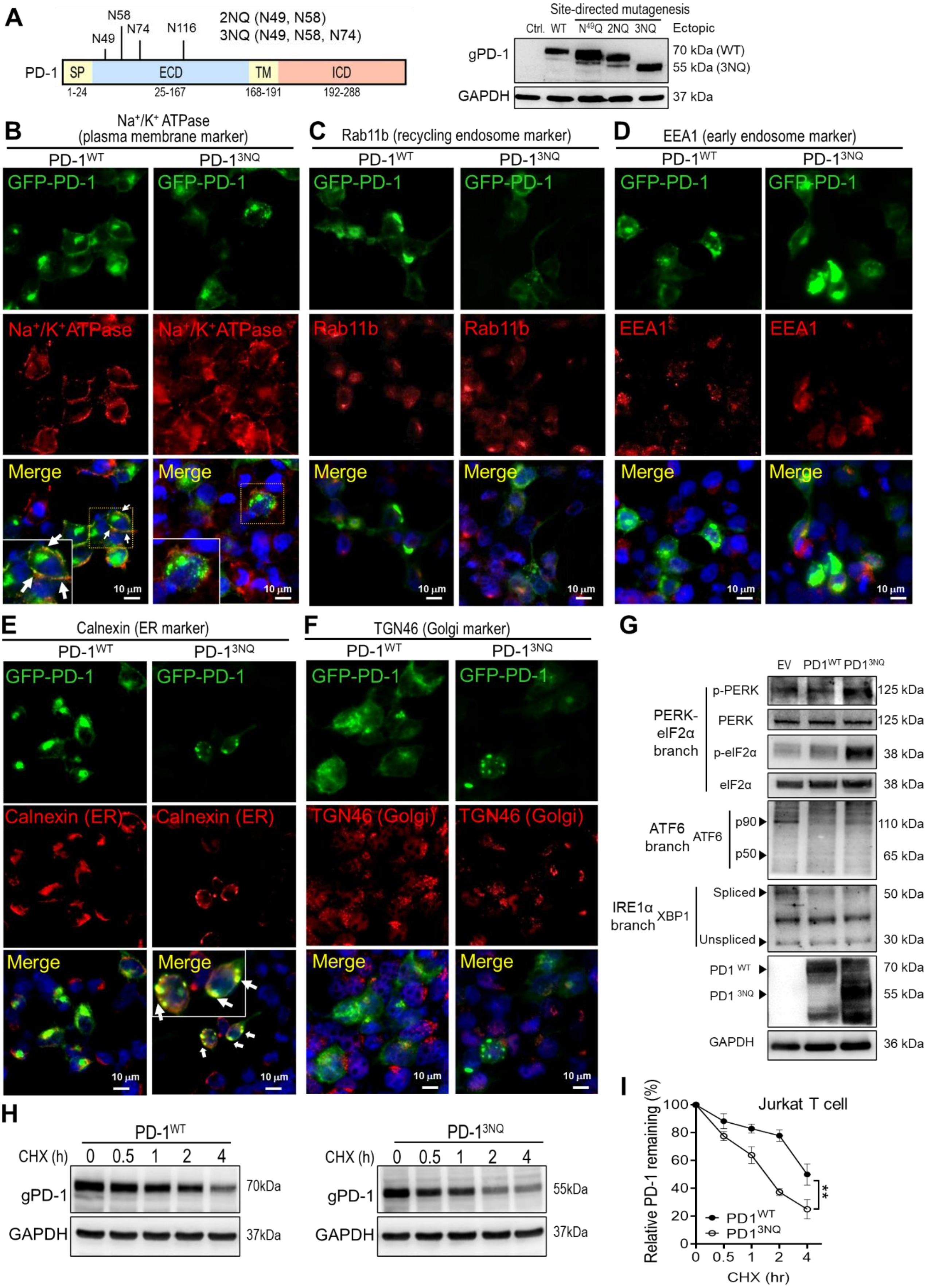

Figure S3. Glycosylation on PD-1 promotes protein stability and transport to the PM. (A) Schematic diagram of full-length PD-1. SP, signal peptide; ECD, extracellular domain; TM, transmembrane domain; ICD, intracellular domain. Four putative Asn-X-Ser/Thr motifs in the ECD domain are labeled in red. The numbers indicate the amino acid positions. We established

glycosylated mutants of PD1 by site-directed mutagenesis. Immunoblot analysis of glycosylated PD-1 (gPD-1) protein expression in WT-PD-1 and NQ-PD-1 Jurkat T cells after transfection for 18 h. **(B-F)** Transfection with GFP-WT-PD-1 and GFP-3NQ-PD-1 in 293T cells for 18 h followed by immunostaining with Na^+/K^+ ATPase, Rab11b, EEA1, Calnexin, and TGN46. **(G)** Western blot analysis of ER stress signaling proteins in COS-1 cells overexpressing GFP-WT-PD1 or GFP-3NQ-PD1. **(H-I)** Jurkat T cells were transfected with GFP-WT-PD1 and GFP-3NQ-PD1 for 18h followed by cycloheximide (CHX, 50 μg/ml) treatment. The quantification of remaining PD-1 protein is shown. The data are shown as the mean ± S.D. P values were determined by two-tailed Student's t test. ** P<0.01

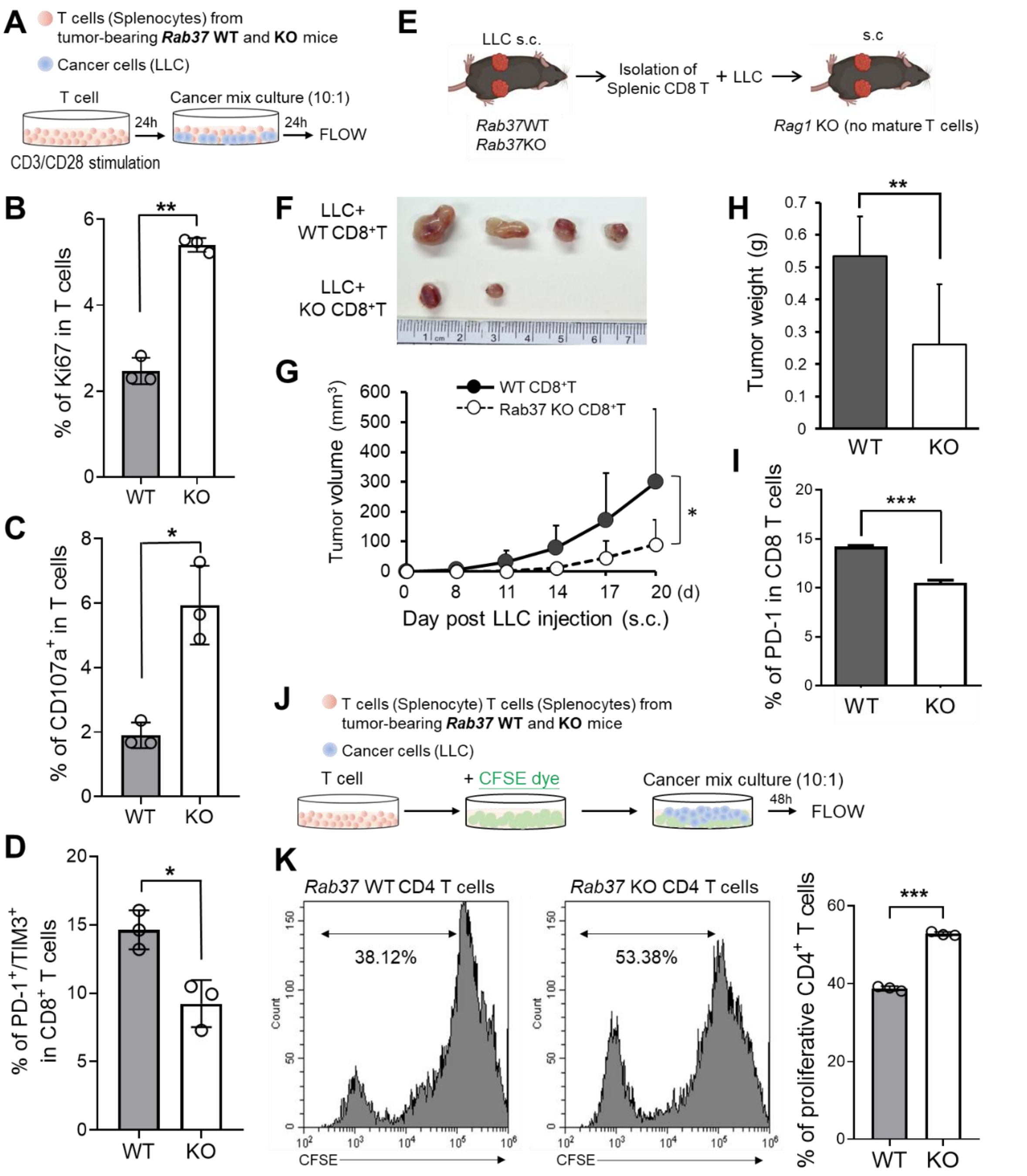

Figure S4. **Rab37-mediated PD-1 PM presentation reduces T cell function. (A-D)** Splenocytes of LLC tumor-bearing *Rab37* KO and WT mice were stimulated for 24 h and/or co-cultured with LLC for another 24 h (A) to analyze the percentage of Ki67^{+} cells (B), cytotoxic-activity marker CD107a expression (C), and PD-1^{+}TIM3^{+} expression (D) by flow cytometry. **(E-I)** Splenic CD8 T cells were isolated from LLC tumor-bearing mice from WT or *Rab37* KO background, mixed with LLC, and then injected subcutaneously (s.c.) into *Rag1* KO mice (E). The tumor size and volume (F-G) and tumor weight (H) were analyzed. The percentages of PD-1^{+} in CD8 T cells derived from WT or *Rab37* KO mice were determined by flow cytometry (I). **(J-K)** Histograms and quantification showing intensity of CFSE on CD4^{+} cells of splenocytes from tumor-bearing *Rab37* WT or KO mice upon stimulation by PMA/Io analyzing for cell proliferation by flow cytometry. The data are shown as the mean ± S.D. P values were determined by two-tailed Student's t test. * *P*<0.05, ** *P*<0.01, *** *P*<0.001.

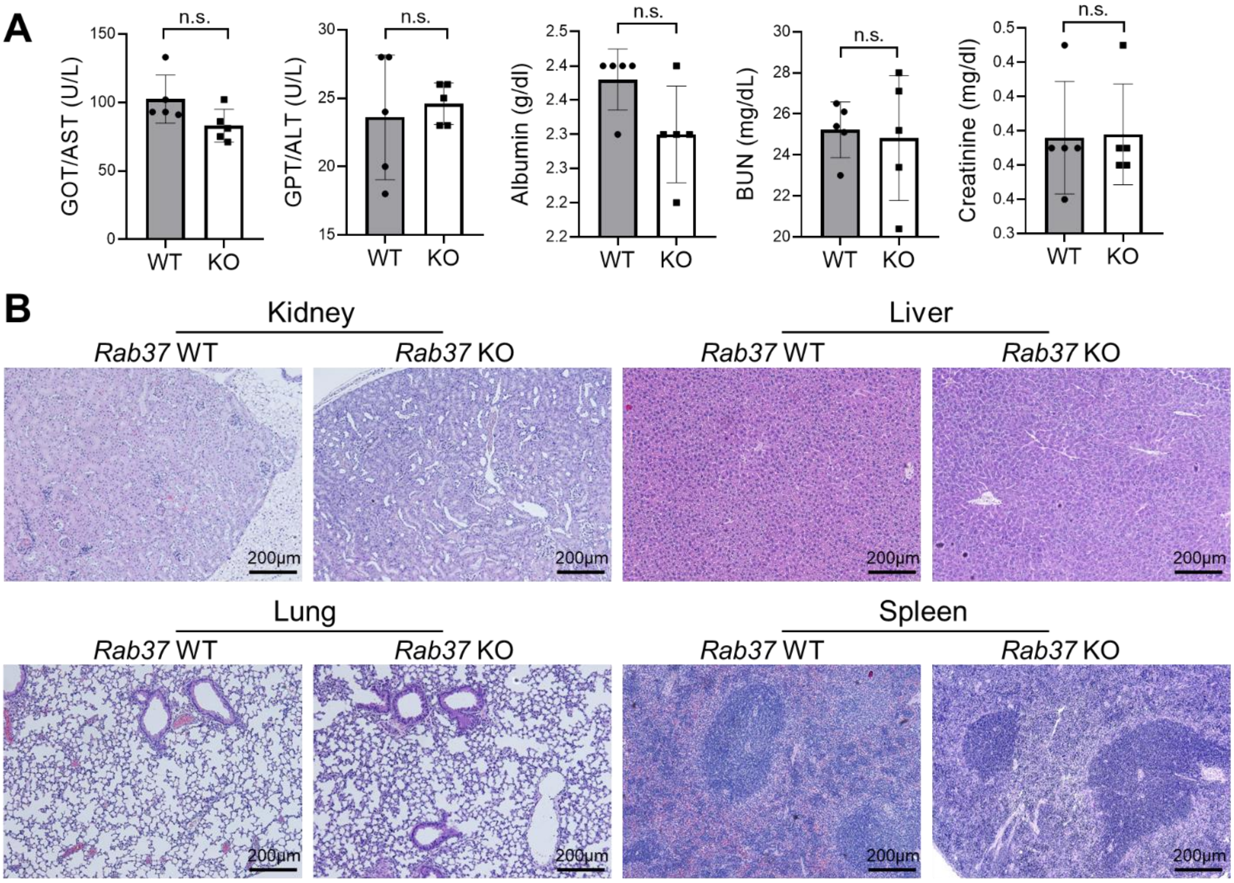

Figure S5. The serum biochemical parameters and major organ histology in LLC tumor-bearing *Rab37* KO and WT mice. (A-B) Serum biochemical markers (A) and major organ histology (B) of treated mice examinations revealed no significant adverse effects between *Rab37* KO and WT mice. Scale bar: 200 μm. The data are shown as the mean ± S.D. P values were determined by two-tailed Student's t test. * *P*<0.05, ** *P*<0.01, *** *P*<0.001.

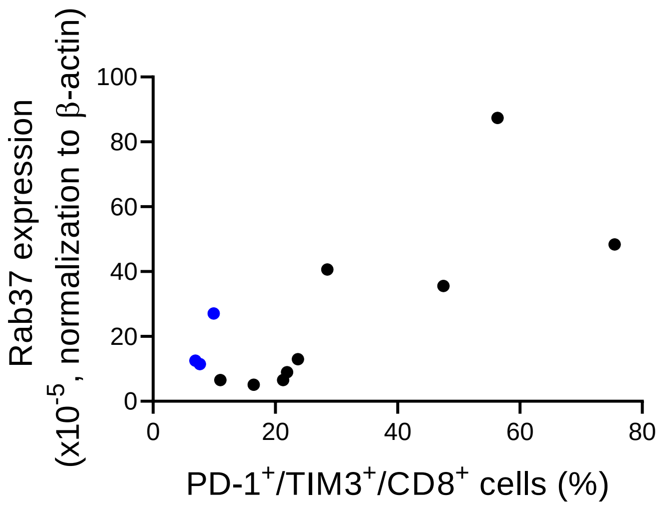

Figure S6. **The relationship between Rab37 expression and population of PD-1/TIM3/CD8 cells derived from PBMCs treated with CD3 and CD28 antibodies.** Blue dot represents PBMCs from healthy donor (N=3). Black dot represents PBMCs from lung cancer patients (N=9).

Movie legends

Movie S1. Time-lapse movie of TIRF images in 293T cells expressing GFP-PD-1 and RFP-tagged Rab37. Images were captured with TIRF microscope at 491 and 561 nm laser every 6 s over a period of 18 s. Time intervals in minutes and seconds are shown. Stills presenting in 00:01 to 00:04 of this movie correspond to 00:00 to 00:18 in **Figure 2F** (RFP-Rab37/GFP-PD-1). Scale bars: 20 µm.

Movie S2. Time-lapse movie of TIRF images in 293T cells expressing GFP-PD-1 with RFP-tagged EV. Images were captured with TIRF microscope at 491 and 561 nm laser every 10 s over a period of 30 s. Time intervals in minutes and seconds are shown. Stills presenting in 00:01 to 00:08 of this movie correspond to 00:00 to 00:30 in **Figure 2G** (RFP-EV/GFP-PD-1). Scale bars: 20 µm.

Movie S3. Time-lapse movie of confocal images in 293T cells expressing GFP-PD-1 and RFP-tagged Rab37. Images were captured with live confocal fluorescence microscope at 491 and 561 nm laser every 10 s over a period of 50 s. Time intervals in minutes and seconds are shown in **Figure S2C** (RFP-Rab37/GFP-PD-1). Scale bars: 10 µm.

Movie S4. Time-lapse movie of confocal images in 293T cells expressing GFP-PD-1 with RFP-tagged EV. Images were captured with live confocal fluorescence microscope at 491 and 561 nm laser every 10 s over a period of 50 s. Time intervals in minutes and seconds are shown in **Figure S2D** (RFP-EV/GFP-PD-1). Scale bars: 10 µm.
